# Proposal for a revised classification of the Demospongiae (Porifera)

**DOI:** 10.1186/s12983-015-0099-8

**Published:** 2015-04-01

**Authors:** Christine Morrow, Paco Cárdenas

**Affiliations:** Queen’s University Belfast, Marine Laboratory, Portaferry BT22 1PF, Northern Ireland, UK; Department of Organismal Biology, Division of Systematic Biology, Evolutionary Biology Centre, Uppsala University, Norbyvägen 18D, 752 36 Uppsala, Sweden; Department of Medicinal Chemistry, Division of Pharmacognosy, BioMedical Centre, Husargatan 3, Uppsala University, 751 23 Uppsala, Sweden

**Keywords:** Taxonomy, Systematics, Sponges, Lithistids, Heteroscleromorpha, Polyphyletic, Monophyletic, Type taxon

## Abstract

**Background:**

Demospongiae is the largest sponge class including 81% of all living sponges with nearly 7,000 species worldwide. *Systema Porifera* (2002) was the result of a large international collaboration to update the Demospongiae higher taxa classification, essentially based on morphological data. Since then, an increasing number of molecular phylogenetic studies have considerably shaken this taxonomic framework, with numerous polyphyletic groups revealed or confirmed and new clades discovered. And yet, despite a few taxonomical changes, the overall framework of the *Systema Porifera* classification still stands and is used as it is by the scientific community. This has led to a widening phylogeny/classification gap which creates biases and inconsistencies for the many end-users of this classification and ultimately impedes our understanding of today’s marine ecosystems and evolutionary processes. In an attempt to bridge this phylogeny/classification gap, we propose to officially revise the higher taxa Demospongiae classification.

**Discussion:**

We propose a revision of the Demospongiae higher taxa classification, essentially based on molecular data of the last ten years. We recommend the use of three subclasses: Verongimorpha, Keratosa and Heteroscleromorpha. We retain seven (Agelasida, Chondrosiida, Dendroceratida, Dictyoceratida, Haplosclerida, Poecilosclerida, Verongiida) of the 13 orders from *Systema Porifera*. We recommend the abandonment of five order names (Hadromerida, Halichondrida, Halisarcida, lithistids, Verticillitida) and resurrect or upgrade six order names (Axinellida, Merliida, Spongillida, Sphaerocladina, Suberitida, Tetractinellida). Finally, we create seven new orders (Bubarida, Desmacellida, Polymastiida, Scopalinida, Clionaida, Tethyida, Trachycladida). These added to the recently created orders (Biemnida and Chondrillida) make a total of 22 orders in the revised classification. We propose the abandonment of the haplosclerid and poecilosclerid suborders. The family content of each order is also revised.

**Summary:**

The deletion of polyphyletic taxa, the use of resurrected or new names for new clades and the proposal of new family groupings will improve the comparability of studies in a wide range of scientific fields using sponges as their object of study. It is envisaged that this will lead to new and more meaningful evolutionary hypotheses for the end-users of the Demospongiae classification.

## Background

The *Systema Porifera* (SP) [1] was the result of a collaboration of 45 researchers from 17 countries led by editors J. Hooper and R. W. M. van Soest. This milestone publication in 2002 provided an updated comprehensive overview of sponge (Porifera) systematics, the largest revision of this group (from genera, subfamilies, families, suborders, orders and class) since the start of spongiology in the mid-19th century. Because before 2002 only a handful of sponge molecular studies were available, the classification of SP is largely based on sponge morphology and re-evaluation of type material, thus providing “a sound platform for the future development of sponge systematics”. Since then, an increasing number of molecular phylogenetic studies have considerably shaken the taxonomic framework of SP (for a review, see [2]) especially concerning the Demospongiae. This is the largest class and includes about 81% of all living sponges with nearly 7,000 species and more than 50 new species on average described every year [3,4].

One of main reasons that molecular results contradict traditional taxonomy may be that this classification was essentially based on the morphology and arrangement of spicules, characters which have repeatedly been shown to be highly homoplasic in demosponges (i.e. prone to convergent evolution and secondary loss) [5-8]. 13 years after SP, the various molecular studies have greatly challenged the Demospongiae classification, telling a largely congruent story where numerous polyphyletic groups have been revealed or confirmed and new clades have been identified. And yet, despite a few taxonomic changes (see Cárdenas et al. [3], p. 159 for a review of these changes), the overall framework of SP classification still stands and is mirrored in the World Porifera Database (WPD, http://www.marinespecies.org/porifera). This is the most widely used reference for sponge nomenclature and part of the World Register for Marine Species (WoRMS). This has led to a widening phylogeny/classification gap which creates biases and inconsistencies for the many end-users of this classification (biochemists, microbiologists, ecologists, conservationists, paleontologists, developmental biologists) and ultimately impedes our understanding of today’s marine ecosystems and evolutionary processes. In an attempt to bridge this phylogeny/classification gap, the studies of Morrow et al. [5,9] in particular, but also Redmond et al. [10] and the review of Cárdenas et al. [3] started to anticipate and suggest a revised higher taxa classification of Demospongiae. Indeed, we still have too few demosponge taxa sequenced to generate a full revision of Demospongiae classification to genus level, but we do have enough taxon coverage to suggest revisions of the higher taxa. The last sentence of the SP preface [1] states: “The *Systema Porifera* project is not an end – but a sound beginning for this new generation to build on what we propose here”. We consider it timely to build on the SP classification and officially propose a revised classification of Demospongiae.

## Discussion

### Revising the classification

**Figure 1:**
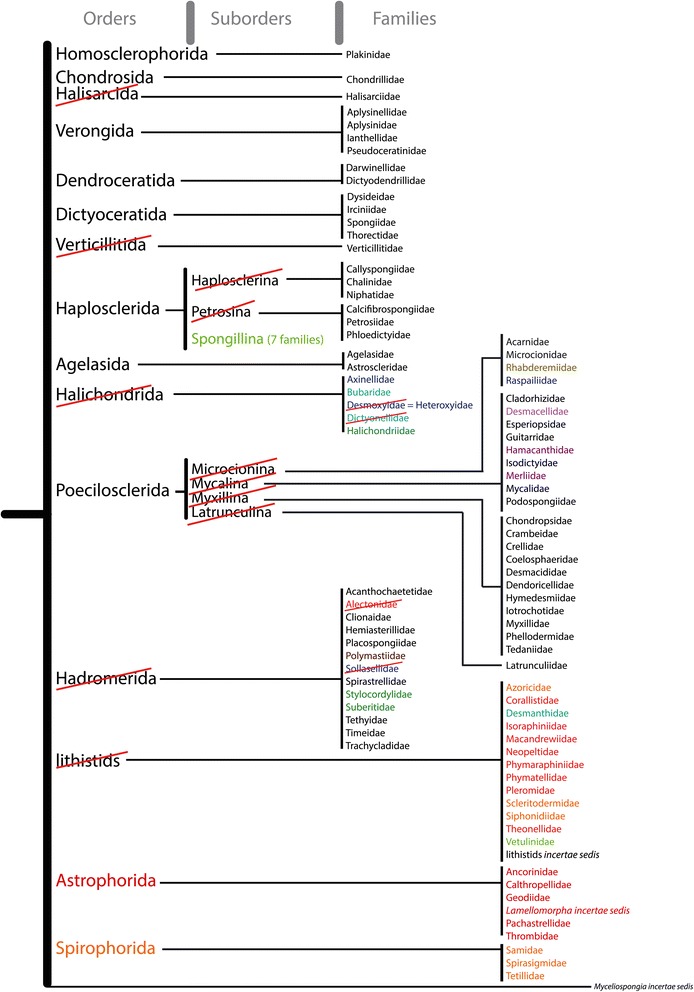
**Demospongiae classification from orders to families, as presented in the** ***Systema Porifera*** **[** 1 **]** **.** Names crossed out in red should be abandoned. Coloured names highlight taxa that should be reallocated; for their new allocation, see Figure 2.

**Figure 2:**
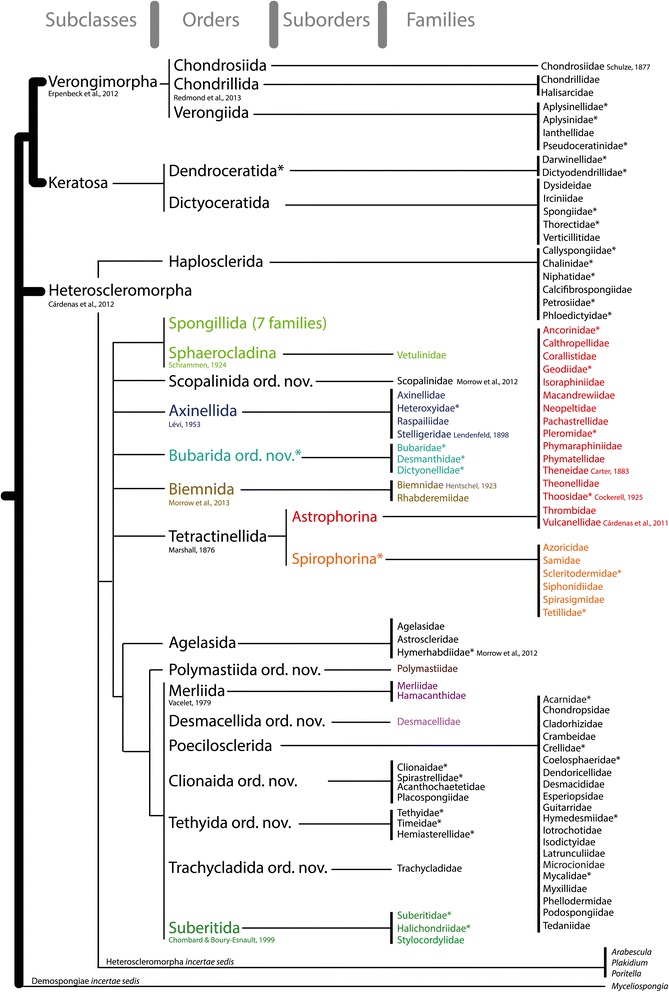
**Proposal for a revised classification of the Demospongiae, from subclasses to families.** Relationships between the different taxa is deduced from all molecular phylogenetic studies published so far (as of November 2014). Coloured names correspond to the same colour code used in Figure 1. Only the authorships of new taxa or resurrected taxa since the publication of the *Systema Porifera* (2002) are given. An asterisk (*) is placed next to all order and family names suspected to be non-monophyletic, based on molecular phylogenetic results (see text for references).

The SP questioned the validity of the subclasses Ceractinomorpha and Tetractinomorpha [11], based on different reproductive strategies. Since then, the polyphyly of the Ceractinomorpha and Tetractinomorpha has been repeatedly confirmed by molecular data. Instead, four well separated Demospongiae clades were identified, often designated under the G1, G2, G3 and G4 clades *sensu* Borchiellini et al. [12]. Since then, these four clades have been considered subclasses and have been named: Keratosa (G1), Myxospongiae (=Verongimorpha) (G2), Haploscleromorpha (G3) and Heteroscleromorpha (G4) [3]. Ceractinomopha and Tetractinomorpha are now officially unaccepted by WPD, but the new four subclasses are not currently implemented in WPD. In this paper, we essentially revisit the current demosponge subclasses, orders and suborders by i) highlighting polyphyletic taxa and the corresponding names that should be abandoned, ii) creating new orders for the newly identified clades and iii) reallocating families to what we believe is their correct order. Doing so, we propose a revised classification of Demospongiae, essentially based on the latest molecular results. To fit the Linnaean rank-based nomenclature, seven new orders (all within the Heteroscleromorpha) have been created to accommodate new groupings of families: Bubarida ord. nov., Desmacellida ord. nov., Polymastiida ord. nov., Scopalinida ord. nov., Clionaida ord. nov., Tethyida ord. nov., and Trachycladida ord. nov.. Other orders not present in SP are upgraded from SP suborders (Spongillida) or resurrected (Axinellida, Merliida, Suberitida, Tetractinellida). Seven SP orders are maintained (Agelasida, Chondrosiida, Dendroceratida, Dictyoceratida, Haplosclerida, Poecilosclerida, Verongiida) and two recently created orders are also included (Chondrillida, Biemnida). Although the naming of orders is not governed by the International Code of Zoological Nomenclature (ICZN), the tradition is to follow a similar rule as for the naming of families (ICZN articles 29.1 and 29.2): adding the suffix –ida to the stem of a genus name (P. Bouchet, pers. comm.). When the genus’ stem ends in –ia, this makes an order name ending in –iida which explains why we decided to modify the names of Verongiida and Chondrosiida from their original spelling with one ‘i’. We have revised the diagnoses of resurrected orders or orders whose content has changed, by revisiting their morphological and chemical characters. We are well aware that some new definitions might appear too wide, due to the fact that we currently lack morphological synapomorphies for these new clades. For taxa where the morphological characters are ambiguous and molecular data are lacking we have used the qualifier ‘*incertae sedis’* when allocating them to a particular higher taxa. To avoid the creation of ‘orphan’ taxa and in order to anticipate the genera re-allocations that will ensue from this proposal, we include a table of Heteroscleromorpha genera (Appendix) with tentative order and family allocations within the framework of our proposal, based on SP and molecular results. We have highlighted where there is supporting molecular data for this allocation and particularly where there is molecular data for the type taxon (Appendix). The allocations of some of the genera are likely to change in the future but we consider this table as a working hypothesis and the necessary first step for the future revision of sponge families and genera. Because of a lack of combined morphological/molecular approaches in Keratosa, Verongimorpha and Haplosclerida *sensu stricto*, the genera content of their families remains to this day unchanged (and is therefore not reviewed in Appendix). Figure 1 represents the *Systema Porifera* Demospongiae classification. Crossed out in red are names that should be abandoned. Figure 2 represents our proposal for a revised Demospongiae classification. Relationships reflect the current knowledge of molecular phylogenetics, resulting from markers *18S*, *28S*, *CO1* (*cytochrome oxidase subunit 1*, usually the Folmer fragment) and almost complete mitochondrial (mt.) genomes. In Figure 2, we have also flagged with an asterisk ‘*’ all the families that are suspected to be non-monophyletic in order to help future systematic studies target problematic groups in need of revision and alert end-users to where contradictory results may arise.

### Three versus four subclasses

One of the main discordant points among sponge taxonomists and the higher taxa may be this one: should we create three subclasses (Verongimorpha, Keratosa and Heteroscleromorpha — including the Haplosclerida) or four (Verongimorpha, Keratosa, Haploscleromorpha and Heteroscleromorpha)? The four subclasses classification originates from the first Demospongiae molecular study which named four distinct clades: G1 to G4 [12]. So the issue is whether marine Haplosclerida can be considered part of the Heteroscleromorpha or not and for this, we should first look at molecular studies with the widest taxon sampling which are those issuing from the Porifera Tree of Life (PorToL) project [10,13], and then at those with the highest number of characters; the mt. genome studies of Lavrov et al. [14]. *18S* suggests there are four clades with strong support (>90 bootstrap support (b.s.)), marine Haplosclerida and Heteroscleromorpha group with moderate support (70 b.s.) [10]. *28S* and mt-genome phylogenetic analyses also find the same four strongly supported clades but this time marine Haplosclerida and Heteroscleromorpha group with a stronger support of 90 b.s. [13-15]. So current molecular data support either three or four subclasses. But skeleton morphology favours three subclasses since Verongimorpha and Keratosa do not have (for the most part) siliceous spicules, and especially do not share the diversity of microscleres present in Heteroscleromorpha and Haplosclerida. By choosing three subclasses we can restrict the order Haplosclerida to the marine Haplosclerida and include it in the Heteroscleromorpha, which becomes by far the largest Demospongiae subclass.

### Deleting polyphyletic groups

#### Abandoning the subclasses Tetractinomorpha and Ceractinomorpha

Tetractinomorpha and Ceractinomorpha are a legacy from the works of Lévi [16,17] that tentatively grouped sponges according to their modes of reproduction (oviparous vs. ovoviviparous). Although early morphological cladistic analysis suggested the polyphyly of these subclasses [18,19], SP followed the classification of Lévi [20] and subdivided the class Demospongiae into three subclasses: Tetractinomorpha, Ceractinomorpha and Homoscleromorpha. The Homoscleromorpha will not be considered here as it was removed from the Demospongiae and is now accepted as a separate sponge class [21]. Shortly after the publication of SP, molecular studies confirmed the polyphyly of Tetractinomorpha and Ceractinomorpha [12,22]. The abandonment of Tetractinomorpha and Ceractinomorpha was officially agreed upon during the 7th International Sponge Symposium (Búzios, Brazil, May 2006), formally published by Boury-Esnault [23] and implemented in WPD.

#### Abandoning Halisarcida

In SP, Chondrosiida includes four genera: *Chondrilla*, *Thymosia*, *Thymosiopsis* and *Chondrosia*. Molecular results have repeatedly suggested the polyphyly of this order with *Chondrilla*, *Thymosia* and *Thymosiopsis* grouping with *Halisarca* (only genus of Halisarcidae, and of Halisarcida) [10,12,24]. Meanwhile *Chondrosia* was either sister group of Verongiida (very well supported) with ribosomal nuclear markers [10,12] or sister-group of a Verongiida + Chondrillidae clade with *CO1* [25]. It was already suggested that Halisarcida should be abandoned and Halisarcidae reallocated to Chondrosiida [26], a proposal also previously made on morphological grounds [27]. The family Chondrillidae Gray, 1872 (including *Chondrilla*, *Thymosia* and *Thymosiopsis*) was resurrected to be associated with Halisarcidae in the new order Chondrillida [10]. Despite contradictory results (*18S*-*28S* vs. *CO1*) with respect to the position of *Chondrosia*, Redmond et al. [10] decided to abandon Chondrosiida and include the resurrected Chondrosiidae Schulze, 1877 in Verongiida. Given the inconsistencies between ribosomal markers and *CO1* with respect to the position of *Chondrosia* we have decided to retain Chondrosiida with its single family Chondrosiidae and its single genus *Chondrosia*.

#### Abandoning Verticillitida

The calcified sponge order Verticillitida contains a single family Verticillitidae with a single living species: *Vaceletia crypta* (Vacelet, 1977). This species has no obvious morphological affinities with any Demospongiae taxa [28]. However, ribosomal and complete mitochondrial data [29,30] suggested that *V. crypta* belonged to Dictyoceratida, making it the only Keratosa sponge with a mineral skeleton. In the WPD, the order Verticillitida has therefore been synonymized with Dictyoceratida and Verticillitidae has been included in Dictyoceratida. One should note that there are still discussions on whether *V. crypta* is related to the fossil family Verticillitidae [28,31]; it has been proposed to classify *V. crypta* in Vacelitiidae Reitner and Engeser, 1985 instead [31,32].

#### Abandoning Spongillina, Haplosclerina and Petrosina

In SP, Haplosclerida includes the following three suborders: Haplosclerina, Petrosina (both marine) and Spongillina (freshwater sponges) (Figure 1). The worldwide monophyletic Spongillina has been upgraded to order rank [3], since molecular results (using mt. genomes, *18S* and *28S*) do not support its grouping with marine Haplosclerida (e.g. [10,13,14]). The only two phylogenetic studies disagreeing with these groupings used seven nuclear housekeeping genes (NHKG). In these two studies, the Spongillida group with the marine Haplosclerida, albeit with either no significant or relatively low support (74 b.s.) [24,33]. Furthermore, the taxonomic sampling in these studies was limited: only two species of Spongillida and no species of Vetulinidae — which may be the sister group of Spongillida [10,34] — and therefore a key group that could considerably alter the topology of the NHKG trees. For the rest of Haplosclerida, often referred to as ‘marine Haplosclerida’, Cárdenas et al. [3] (p. 170) proposed the subclass name Haploscleromorpha. Here we abandon Haploscleromorpha and retain Haplosclerida (with a revised definition) as an order within the subclass Heteroscleromorpha (cf. discussion above on ‘Three versus four subclasses’).

Although Haplosclerida is a well-supported clade, the suborders Haplosclerina and Petrosina and almost every family therein appear polyphyletic (for a review see [3], p. 192; [10]). A revision of Haplosclerida using new character datasets and implementing a bottom-up approach, studying first the type species of each genus [3] within each of the 5 newly found clades — provisionally called clade A to E [10] — is urgently needed. In the meantime, we propose to abandon these suborders.

#### Abandoning Lithistida

Lithistida Schmidt, 1870 had been considered an artificial and polyphyletic group long before it was confirmed by molecular results. The only shared character of Lithistida is interlocked spicules called ‘desmas’ which form a rigid skeleton. Lithistida are easily fossilized and thus have an extremely rich fossil record in comparison with other Demospongiae. Despite their acknowledged polyphyly and after numerous debates during the SP genesis, desma-bearing demosponges were grouped together under the name “lithistid’ Demospongiae”, mainly for convenience (Figure 1). Even though SP proposed to abandon the order Lithistida [35], this name has remained in the WPD, which can be very misleading for end-users such as biochemists, microbiologists, ecologists or paleontologists. Now that we have molecular support concerning the phylogenetic affinities of most of the desma-bearing families [6,36], we propose to formally reallocate the 13 desma-bearing families to their respective Heteroscleromorpha orders, as was already done by Cárdenas et al. [3,6], and abandon the Lithistida name in WPD. 11 out of the 13 desma-bearing families should be moved to the Tetractinellida: 8 families to the Astrophorina, 3 families to the Spirophorina. The Vetulinidae are now moved to their own order Sphaerocladina (an existing order in the fossil classification) and the Desmanthidae are allocated to Bubarida ord. nov.. ‘Lithistids *incertae sedis*’ from the SP (*Arabescula, Plakidium* and *Poritella*) should now be referred to as ‘Heterosclermorpha *incertae sedis*’ (see below).

#### Abandoning Poecilosclerida suborders: Microcionina, Mycalina, Myxillina and Latrunculina

**Figure 3:**
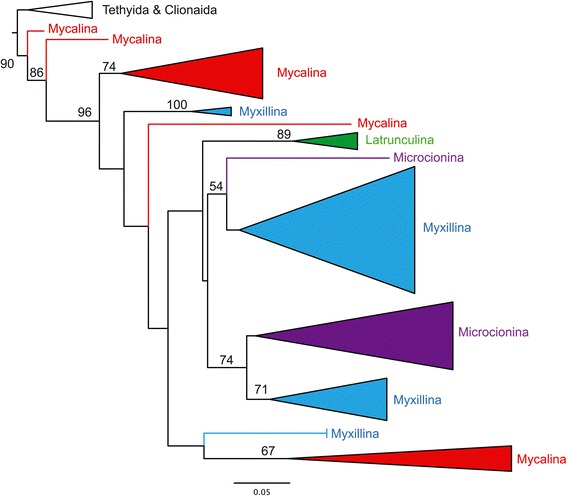
***18S*** **tree revealing the polyphyly of the Poecilosclerida suborders.** PhyML tree with branches collapsed showing the polyphyly of the poecilosclerid suborders (Microcionina, Mycalina, Myxillina), with the exception of Latrunculina; only node bootstrap support > 50 are shown. This is a subset of the data used in Redmond et al. [10].

In SP, Poecilosclerida comprised 25 families, distributed in four suborders erected by Hajdu et al. [37]: Microcionina, Mycalina, Myxillina and Latrunculina (Figure 1). These suborders essentially rely on the presence/absence and morphology of chelae microscleres. The SP classification is based on the assumption that chelae can be used to reconstruct phylogeny because of their morphological complexity and presumed selective neutrality but it seems that convergent evolution has brought phylogenetic noise to this hypothesis. Although we are far from understanding the phylogenetic relationships within this large order, molecular studies (using *CO1*, *28S* and *18S*) strongly suggest that Microcionina, Mycalina and Myxillina are polyphyletic (Figure 3) [7,10,13,38]. We therefore propose to abandon these suborder names. Latrunculina, which only includes Latrunculiidae, seems to be monophyletic [10,38] but for consistency it is here abandoned along with the other suborders. It is not possible to provide an alternative internal phylogenetic structure for Poecilosclerida since so few taxa have yet been sequenced.

#### Abandoning Halichondrida

The taxonomic history of this group is long and complex (for a review, see [39]). The SP order Halichondrida contains the following five families: Halichondriidae, Axinellidae, Dictyonellidae, Heteroxyidae and Bubaridae (Figure 1). However, the monophyly of Halichondrida has never been recovered in any morphological, molecular or biochemical cladistics analyses (e.g. [5,39,40]). Halichondrida lack any unambiguous synapomorphic characters and are mainly defined on the basis of shared negative characters. Using the *28S* rDNA marker, Chombard [41] and Chombard & Boury-Esnault [42] first revealed a close relationship between Halichondriidae and Suberitidae and not with other families assigned to Halichondrida. Chombard & Boury-Esnault [42] proposed the name Suberitina for this new clade. This clade was consistently confirmed in subsequent molecular phylogenetic studies, using more taxa and additional markers (e.g. [5]) and we now consider it should be upgraded to the order rank as Suberitida. At the same time, the other Halichondrida families were distributed amongst other clades: Axinellidae and Heteroxyidae in a well-supported clade for which we use the resurrected order name Axinellida; Dictyonellidae and Bubaridae in another clade here named Bubarida ord. nov. (Figure 2). Finally, *18S* and *CO1* data revealed a new clade (unnamed at this moment) grouping species of *Topsentia*, *Petromica* and *Axinyssa* [10,36]. Altogether, these well-established results force us to formally propose the abandonment of Halichondrida.

#### Abandoning Hadromerida

**Figure 4:**
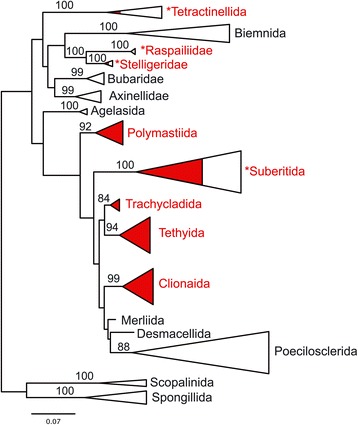
***18S*** **tree revealing the polyphyly of Hadromerida.** PhyML tree with branches collapsed showing polyphyly of Hadromerida (shown in red) that we propose to abandon; only nodes with > 50 b.s. are shown. This is a subset of the data used in Redmond et al. [10]. *Raspailiidae, *Suberitida, *Stelligeridae and *Tetractinellida include a mix of former ‘hadromerid’ taxa as well as taxa from other orders.

Hadromerida in SP included 13 families (Figure 1), two of which have now been abandoned: Alectonidae (split between Astrophorina and Clionaidae) and the Sollasellidae (now a junior synonym of Raspailiidae). Suberitidae has often been considered as a typical ‘hadromerid’. However, since the work of Chombard & Boury-Esnault [42], Suberitidae have been shown to group with Halichondriidae in a well-supported clade. A *CO1* sequence of *Stylocordyla borealis* suggests that Stylocordylidae, which was also considered a ‘hadromerid’, groups with Suberitidae and Halichondriidae (Morrow and Cárdenas, unpublished results). On the basis of *28S* rDNA data, Chombard [41] had anticipated that the remaining ‘hadromerids’ grouped in four well-supported clades, later confirmed with larger sampling and additional markers. One contains Spirastrellidae, Clionaidae, Placospongiidae and Acanthochaetetidae; a second Timeidae, Tethyidae and Hemiasterellidae (pars); a third Trachycladidae and a fourth Polymastiidae [5,9,10,13]. Figure 4 is an *18S* ML tree which shows the distribution of former SP ‘hadromerid’ taxa relative to other Heteroscleromorpha. Lavrov et al. [14] using mitochondrial genomes showed Tethyidae grouping separately to Clionaidae but his analysis did not include Trachycladidae. Some former Hemiasterellidae have also joined some former halichondrids to group in the resurrected Stelligeridae family [5]. Altogether, given the polyphyly of Hadromerida (Figure 4) we propose the abandonment of Hadromerida, the erection of four new orders (Clionaida ord. nov., Tethyida ord. nov., Trachycladida ord. nov., Polymastiida ord. nov.) and the elevation of Suberitina to Suberitida Chombard & Boury-Esnault, 1999.

### Taxonomy and definitions

For the family composition of each order, see Figure 2. For a tentative generic composition of the orders, see Appendix.

### Subclass Verongimorpha Erpenbeck et al., 2012

Definition: Demospongiae without skeleton or with a skeleton made of siliceous asters (*Chondrilla*) or spongin fibres with a laminated bark and a finely fibrillar or granular pith (most of the Verongiida and *Thymosia*). Epithelial cells of the larva have i) a non-perpendicular orientation of the accessory centriole relative to the basal body, ii) a protruding nuclear apex and iii) a Golgi apparatus around the nuclear apex and part of the organelles of the basal apparatus (definition from [3], emended with larva observations from [43]).

Remark: We add here larva cytological characters in the definition but underline that very few Verongimorpha larva have been studied so far [43] so these characters need to be confirmed. Borchiellini et al. [12] resurrected Myxospongiae for their G2 clade (*Halisarca* + *Chondrosia* + Chondrillidae + Verongiida); since then, the name has been used and even erected as a subclass [43]. But Myxospongiae was originally intended for sponges without any skeleton (“*ohne jedes Skelet*” [44]), the so called “slime sponges” by Haeckel [44], which was essentially *Halisarca*. Erpenbeck et al. [25] consider that the G2 clade assemblage is too different from the original content of Myxospongiae and decide to create a replacement name for the Myxospongiae: Verongimorpha. Erpenbeck et al. [25] also consider that most of the sponges in this group are not “slime-sponges” so that the name of the subclass is not descriptive enough of the group. Myxospongiae *sensu* Haeckel, 1866 has been seldom used in the past and so, despite the different assemblage of Myxospongiae (G2), we believe there has not been confusion: the name Myxospongiae has been properly used now for over ten years. However, we agree that the Myxospongiae name poorly reflects the sponges it contains so we recommend using the name Verongimorpha.

**Order Chondrillida Redmond et al.,** [10]

Definition: Verongimorpha in which the skeleton can be absent, but when present is composed of nodular spongin fibers or aster microscleres [10].

**Order Chondrosiida Boury-Esnault & Lopès, 1985**

Definition: Verongimorpha with a marked ectosome or cortex enriched by a highly organized fibrillar collagen. Collagen is always very abundant. (modified from [3], p. 170).

Remark: This order only includes the genus *Chondrosia*.

**Order Verongiida Bergquist, 1978**

SP definition emended from Bergquist and Cook [45]: Verongimorpha in which the fibrous skeleton, when present, is either anastomosing or dendritic in construction. Reproduction is always oviparous. They produce complex brominated tyrosine-derived compounds.

Remark: Most relationships are uncertain within this order; Pseudoceratinidae, Aplysinellidae and Aplysinidae are probably not monophyletic [10,13,25,46,47].

### Subclass Keratosa Grant, 1861

Definition: Demospongiae with a skeleton made of spongin fibre. Spongin fibres are either homogenous or pithed and strongly laminated with pith grading into bark. One genus has a hypercalcified basal skeleton (*Vaceletia*). ([3], p. 170).

**Order Dendroceratida Minchin, 1900**

SP definition emended from Bergquist and Cook [48]: Keratosa in which a fibre skeleton is always present but, as compared to Dictyoceratida, is reduced in relation to soft tissue volume. The skeleton arises from a continuous spreading basal plate, and adopts either a dendritic or an anastomosing pattern. In anastomosing forms there is never any clear size distinction between primary and secondary elements. The fibres always contain pith and are strongly laminated, usually quite stout, and in some genera cellular (degenerate spongocytes) elements are incorporated in the bark and to a lesser extent in pith. Free fibrous spicules may supplement the main skeleton. The pith in the fibres is markedly disjunct from the bark, and in structure is close to that of the Verongiida. Biochemically, members of this group are characterized by a moderate sterol content in conjunction with the presence of terpenes, which are always diterpenes.

Remark: This order and both of its families (Darwinellidae and Dictyodendrillidae) may be polyphyletic [10,12,25].

**Order Dictyoceratida Minchin, 1900**

SP definition emended from Cook and Bergquist [49]: Keratosa in which a spongin fibre skeleton is constructed on an anastomosing plan. The skeleton develops from multiple points of attachment and, except in two genera where primary fibres are absent, is organized as a hierarchy of primary, secondary and sometimes tertiary elements. Fibre construction is homogeneous lacking pith with growth laminae tightly adherent and just detectable, or pithed and strongly laminated with pith grading into bark, consecutive laminae are marked but remain adherent to each other. Pith is structurally and chemically distinct from that seen in fibres of Verongiida and Dendroceratida. One species (*Vaceletia crypta*) has a ‘sphinctozoan’ grade of organization with a chambered skeleton composed of aragonite with irregular structure, without siliceous spicules.

Remark: The families Spongiidae and Thorectidae are not monophyletic and blend essentially together; some Thorectidae should also be reallocated to the Dysideidae [10,13,25].

### Subclass Heteroscleromorpha Cárdenas et al., [3]

Definition: Demospongiae with a skeleton composed of siliceous spicules which can be monaxons and/or tetraxons and when they are present, microscleres are highly diversified (definition of Cárdenas et al. [3] was not modified by the inclusion of Haplosclerida).

**Order Haplosclerida Topsent, 1928**

Definition: Heteroscleromorpha with an isodictyal anisotropic or isotropic choanosomal skeleton; spicules are diactinal megascleres (oxeas or strongyles), smooth or spined; microscleres, if present, are sigmas and/or toxas, microxeas or microstrongyles (emended definition of Haploscleromorpha Cárdenas et al., [3]).

Remark: Five out of the six families, as well as many genera are not monophyletic [10]. The last family (Calcifibrospongiidae) is monospecific. Currently, five well-supported clades (clades A-E) are recognized [10]. The definition of Cárdenas et al. [3] has been emended here to include *Janulum,* formerly assigned to Raspailiidae, and now included in clade E [10].

**Order Spongillida Manconi & Pronzato,** [50]

SP definition of Spongillina from Manconi and Pronzato [50]: Exclusively freshwater sponges, with megascleres consisting of oxeas or strongyles, smooth or spined, forming pauci- to multispicular tracts producing irregular to regular meshes, occasionally with large alveolate cavities (a central body cavity in one family); spongin mostly sparse; microscleres present or absent, including smooth or spined oxeas, aster-like or birotule like spicules. Four families with gemmules (resting bodies containing totipotent cells), which may contain gemmuloscleres of diverse morphology that is often diagnostic. Three families lack gemmules. Where known, reproduction is viviparous, with fully ciliated parenchymella larvae.

Remark: Malawispongiidae is polyphyletic and some taxa may need to be reallocated to Spongillidae [51]. Based on *CO1*, *18S* and *ITS2* phylogenetic analyses, Spongillidae are not monophyletic [52,53]. The monotypic family Metschnikowiidae is a doubtful Spongillida and may need to be reallocated to the Haplosclerida. Indeed, it is endemic to the Caspian Sea, thus living in brackish water instead of freshwater; its morphological characters suggest possible affinities with *Janulum* [54,55], recently reallocated to Haplosclerida (clade E) [10].

**Order Sphaerocladina Schrammen, 1924**

SP definition of Vetulinidae emended from Pisera & Lévi [56]: Heteroscleromorpha with acrepid polyaxial (astro- or sphaeroclone) desmas. No other megascleres or microscleres, only one recent family but several fossil families.

Remark: We decided to use the paleontological order name since this group has a very long and rich fossil record [56]. A sister-group relationship with Spongillida is strongly supported by *18S* [10,34], *CO1* and *28S* [36].

**Scopalinida ord. nov.**

Definition: Encrusting, massive or erect flabellate growth forms; with smooth or conulose surface supported by prominent spongin fibres cored with styles; megascleres styles, often with telescoped ends; no ectosomal skeleton; tissue contains an unusual cell type filled with refractile granules.

Remark: This order contains only one family, Scopalinidae with genera *Scopalina* and *Svenzea. Stylissa*, a former halichondrid, is here tentatively included in this order/family (Appendix) since the type species *Stylissa flabelliformis* grouped within the Scopalinidae [10].

**Order Axinellida Lévi, 1953 (resurrected)**

Definition: Megascleres are styles or tylostyles and oxea, with acanthostyles in some genera. Surface may be smooth but is usually hispid due to projecting choanosomal styles and these may be surrounded by brushes of fine oxea, anisoxea or styles forming a specialised ectosomal skeleton. Micoscleres when present are asters, acanthoxea or raphides, usually in trichodragmata (emended from [5]). Skeleton in several species comprised of a stiff axial region, usually with abundant spongin and an outer, softer extra-axial region. Colour of living sponge is characteristically orange, yellow or dark brown.

Remark: The order Axinellida Lévi, 1953 was originally erected for the families Axinellidae and Raspailiidae [17]. In subsequent publications Lévi assigned seven other families to this order. Whilst the content of Axinellida has changed from that formally defined by Lévi (1973) [20] and Bergquist (1967; 1970) [57,58], various molecular studies have shown Axinellida to contain three of the nine families that have been assigned to it. Most importantly the studies show that *Axinella polypoides*, the type taxon for Axinellidae clusters here. The principles of the ICZN for family names are based on the name-bearing type genus. Whilst at order level we are not bound by the rules of the ICZN, in keeping with the spirit of the code, we retain the name Axinellida for this order. The Raspailiidae have been moved back from Poecilosclerida to this order based on numerous molecular studies (e.g. [9,10]). The family Stelligeridae was resurrected by Morrow et al. [5] to include former hadromerid (*Stelligera, Paratimea*) and halichondrid genera (*Halicnemia, Higginsia*). Redmond et al. [10] using *18S* rDNA shows strong support for a Stelligeridae-Raspailiidae clade but no support for Stelligeridae-Raspailiidae + Axinellidae. However in Morrow et al. [9] using *18S* + *28S*, Stelligeridae-Raspailiidae + Axinellidae is a strongly supported clade. Unpublished results by C. Morrow show that some *Heteroxyidae* also cluster with Axinellida.

**Order Bubarida ord. nov.**

Definition: Heteroscleromorpha built with monactines, diactines of different kinds and different shapes (flexuous, sinuous or vermiculiform; telescoped endings are common). Flexuous or sinuous spicules may be confined to axial skeleton or form a basal layer in encrusting forms. Monocrepidial desmas form a basal skeleton in one genus (*Desmanthus*).

Remark: This order is in need of taxonomic revision: early indications are that there are three clades mixing species of *Acanthella, Dictyonella, Bubaris, Cymbastella, Axinyssa, Phakellia* and *Phycopsis* [5,10]. In the future it is likely that the family Dictyonellidae van Soest, Diaz & Pomponi, 1990 and Bubaridae Topsent, 1894 will be merged, however Dictyonellidae is retained here as a revision of the order is beyond the scope of this study. It is also possible that older family names such as Desmanthidae Topsent, 1893 may take priority over Bubaridae. The lithistid family Desmanthidae is assigned to this order since the type species *Desmanthus incrustans* joins this clade, based on *18S*, *CO1* and *28S* markers [5,10,59]. However, *CO1* and *18S* studies also suggest that *Petromica* (Desmanthidae), *Topsentia* (Halichondriidae) and some *Axinyssa* (Halichondriidae) group in a very well supported clade (unnamed at the moment) outside of Bubarida [10,36], therefore Desmanthidae and Bubarida are both polyphyletic. For now we retain the family Desmanthidae pending further molecular evidence.

**Order Biemnida Morrow et al., 2013**

Definition: Megascleres styles, subtylostyles, strongyles, rhabdostyles, or oxeas. Spicules typically enclosed by spongin fibres. Reticulate or plumoreticulate choanosomal skeleton, axially compressed in erect forms. Extra-axial plumose skeleton usually present. Microscleres are microspined sigmas/spirosigmas, toxas, microxeas, raphides, and/or commata. *Biemna* and *Neofibularia* cause a dermatitis-like reaction when in contact with bare skin [9].

Remark: This order includes former Desmacellidae genera (*Biemna, Neofibularia* and *Sigmaxinella*), grouped in the resurrected family Biemnidae, as well as the Rhabderemiidae family with a single genus *Rhabderemia.* Grouping of these two families is based on molecular and morphological data, notably the possession of distally microspined “sigmas”, which may not be homologous to the sigmas found in poecilosclerid sponges for example [7,9,10,60,61]. In SP, all these genera were in the Poecilosclerida order since they were believed to have lost their chelae.

**Order Tetractinellida Marshall, 1876 (resurrected)**

Definition: Heteroscleromorpha usually with radial or subradial skeletal arrangement, some genera can be endolithic. Megascleres are monactines and triaenes in various shapes (a synapomorphy of the order, but sometimes secondarily lost). Microscleres include sigmas, asters, sometimes with microrhabds, microxeas and raphides. Desmas are sometimes present.

Remarks: Astrophorida and Spirophorida were included in SP while Tetractinellida was not. However, all molecular studies have shown that Tetractinellida is a strongly supported clade (e.g. [10,62]). We propose to resurrect this order, and recommend the use of the suborders Astrophorina and Spirophorina, as previously suggested [3,5]. Many Astrophorina families and genera seem to be polyphyletic, especially due to the mingling of Ancorinidae and Geodiidae genera [6,63]. Since SP, one family was resurrected (Theneidae) and a new family was created (Vulcanellidae) [6]. Alectonidae, formerly in Hadromerida has been abandoned and the genera *Alectona/Delectona/Thoosa* and *Neamphius* have been artificially kept together in the resurrected Thoosidae, now assigned to Astrophorina, awaiting a clearer appreciation of the phylogenetic relationships of *Neamphius*. The genera *Lamellomorpha* (Astrophorida *incertae sedis* in SP) and *Characella*, are both provisionally assigned to Pachastrellidae, which makes this family polyphyletic [6,10]. Most of the desma-bearing demosponge families have now been assigned to this order: eight and three families are now respectively assigned to Astrophorina and Spirophorina. Studies based on *COI*, *18S* and *28S* suggest that Spirophorina and Tetillidae may not be monophyletic [10,36]. It also seems that Pleromidae and Scleritodermidae may not be monophyletic [36].

**Order Agelasida Hartman, 1980**

Definition: Megascleres smooth or verticillately spined styles, rhabdostyles or ocassionally oxea, no microscleres. Representatives of all families of the order produce similar pyrrole-2-carboxylic compounds, characteristically with a bromine addition [5].

Remark: The new family Hymerhabdiidae added to this order currently includes the former halichondrid *Hymerhabdia* and the former hadromerid *Prosuberites*, in addition to some former halichondrids belonging to *Stylissa*/*Axinella/Phycopsis/Cymbastela* [5,10,64]. The former raspailiid genera *Acanthostylotella* and *Amphinomia* are now assigned to Agelasidae [10].

**Polymastiida ord. nov.**

SP definition by Boury-Esnault [65]: Heteroscleromorpha with a radiating choanoskeleton and a more or less complicated cortex, the outer layer being always a palisade of ectosomal spicules (tylostyles, or oxea and/or exotyles). Megascleres are tylostyles, subtylostyles, strongyloxeas, styles or oxeas; microscleres may include centrotylote microxeas, acanthose microxeas or raphides in trichodragmata.

Remark: This order only includes the family Polymastiidae.

**Order Merliida Vacelet, 1979 (resurrected)**

Definition: Megascleres are diverse (oxeas, styles, mycalostyles or tylostyles) but associated with unique microscleres (either clavidiscs or diancistra-derivatives: diancistras or cyrtancistras). Raphides, sigmas or small commata-like spicules are also present. One family contains a species with a chaetetid calcareous basal skeleton, and an outer layer of which is filled with sponge tissue and siliceous spicules.

Remarks: Vacelet [66,67] first suggested to isolate *Merlia* in its own order — Merliida *incertae sedis* — but in the SP it was included in the Poecilosclerida, based on microscleres similarities with the Biemnidae and Desmacellidae [68]. The recognition of a separate order was actually confirmed in *18S, 28S* and *16S* phylogenetic analyses, where it branches separately from the rest of the Poecilosclerida [10,59]. *CO1* phylogenetic analyses further show that *Hamacantha* and *Desmacella* branch before the poecilosclerid clade (Morrow, unpublished results). Topsent [69] was the first to suggest a close relationship between *Hamacantha* and *Merlia* based on the striking similarity between the diancistras (in *Hamacantha*) and the clavidiscs (in *Merlia*). On the basis of this molecular result and also the strong morphological affinities of the microscleres we propose to move Hamacanthidae from Poecilosclerida to Merliida.

**Desmacellida ord. nov.**

SP definition by Hajdu and van Soest [70]: Heteroscleromorpha with monactinal megascleres arranged in plumose bundles; microscleres sigmas and sometimes raphides.

Remark: Desmacellidae, a family without chelae, seems to diverge before the Poecilosclerida radiation [10] and is here assigned its own order. *Biemna, Neofibularia* and *Sigmaxinella* which were allocated to Desmacellidae in SP, were transferred to Biemnida [9]; the remaining genera, *Desmacella, Dragmatella* and *Microtylostylifer*, are allocated to Desmacellida. However, it should be noted that there is no molecular data for *Dragmatella* and *Microtylostylifer* species.

**Order Poecilosclerida Topsent, 1928**

SP definition emended from Hooper & van Soest [71]: both fibre and mineral skeletons always show regional differentiation such that megascleres are often differentiated into distinct ectosomal and choanosomal components; microscleres include chelae (a synapomorphy for the order, but sometimes lost), sigmas and sigmancistra derivatives, and other diverse forms such as toxas, raphides, microxeas, discorhabds or spinorhabds; the order appears to be exclusively viviparous.

Remark: We propose to abandon the suborders (cf. above and Figure 3). The Poecilosclerida included 25 families in the SP, five of which we now propose to reassign to Desmacellida, Merliida, Axinellida and Biemnida. Possible polyphyletic families include Acarnidae, Mycalidae, Coelosphaeridae, Hymedesmiidae and Crellidae [10,13].

**Clionaida ord. nov.**

Definition: Heteroscleromorpha with tylostyle megascleres; oxeas and styloid spicules are also present in one family. Variety of microscleres including streptasters (spirasters and diplasters), amphiasters, selenasters, microxeas, microrhabds, spiral microstrongyles and derivatives. Microscleres may be lacking altogether. Calcareous basal skeleton present in one family.

Remark: Clionaidae d’Orbigny, 1851 and Spirastrellidae Ridley and Dendy, 1886 seem to mix and may thus not be monophyletic [5,22,72]. Since Clionaidae is an older family name, there is a chance that Spirastrellidae might become invalid in the future so we chose Clionaida over Spirastrellida (which had been suggested by Chombard [41] in her PhD thesis).

**Tethyida ord. nov.**

Definition: Megascleres may be styles, tylostyles or oxeas arranged in tracts ending as bouquets, at or near the surface. Microscleres are euasters, usually of two sizes.

Remark: Morrow et al. [5] showed that Hemiasterellidae is polyphyletic with some genera grouping closely with some heteroxyid and raspailiid taxa and others with Tethyidae. In the absence of molecular data from the type taxon (*Hemiasterella typus*) we retain Hemiasterellidae in Tethyida. Furthermore, Timeidae and Tethyidae may not be monophyletic, but molecular data on these two families is sparse [5,10,22]. We chose the name Tethyida for this order since we have molecular data for the type taxon of Tethyidae (*Tethya aurantium*) but not for the type taxons of Timeidae or Hemiasterellidae.

**Trachycladida ord. nov.**

SP definition of Trachycladidae by Hooper & van Soest [73]: Heteroscleromorpha with spined vermiform spinispirae and smooth microrhabds, with a differentiated axial and extra-axial skeleton cored by oxeas, strongyles and/or (tylo-)styles.

Remark: Only one family (Trachycladidae), with 10 species, belongs to this order at the moment.

**Order Suberitida Chombard & Boury-Esnault, 1999**

Definition: Heteroscleromorpha without an obvious cortex and without microscleres other than microstrongyles/oxeas; megascleres are oxeas, centrotylote oxeas, styles or tylostyles. Choanosomal skeleton usually consisting of a confused arrangement of megascleres, radial arrangement of megascleres in one family. Surface skeleton of paratangential to erect palisade of large or small megascleres. Molecular synapomorphy is a deletion of a small loop of 15 base pairs in the secondary structure of the *28S* D2 domain with respect to other Heteroscleromorpha (slightly emended from [42] to include Stylocordylidae).

Remark: Suberitidae and Halichondriidae are currently not monophyletic due to, for example, the grouping of *Terpios* with the halichondrids instead of the suberitids, or the early branching of *Homaxinella* with respect to the rest of the Suberitida [5,10,13]. The allocation of *Stylocordyla* to Suberitida is based on previous morphological studies (e.g. [74,75]) and molecular data (*CO1*) (Morrow and Cárdenas, unpublished results).

**Heteroscleromorpha*incertae sedis*** may include some of the former ‘lithistids’ *incertae sedis* listed in SP, which consists of poorly known genera with rhizoclone desmas of uncertain status [76]: *Arabescula*, *Plakidium* and *Poritella. Collectella* is clearly a tetractinellid with phyllotriaenes that we tentatively assign to Theonellidae. *Collinella* has been synonymized with *Discodermia* (Theonellidae) in the WPD.

***Myceliospongia*** with its single species *M. araneosa* remains Demospongiae *incertae sedis*, as in the SP [77], awaiting molecular data to assign it to an order.

### Concluding remarks

Demospongiae in SP is comprised of 13 orders. In the present proposal, five of these order names are abandoned (Hadromerida, Halichondrida, Halisarcida, lithistids, Verticillitida) and six order names are resurrected or upgraded (Axinellida, Merliida, Spongillida, Sphaerocladina, Suberitida, Tetractinellida) and seven new orders have been erected (Bubarida, Desmacellida, Polymastiida, Scopalinida, Clionaida, Tethyida, Trachycladida). These added to the recently created orders (Biemnida and Chondrillida) make a total of 22 orders in the revised classification. We propose the abandonment of all Haplosclerida and Poecilosclerida suborders and the use of Tetractinellida suborders. Finally, we reassign many families (belonging to Hadromerida, Halichondrida, Halisarcida, Poecilosclerida, Lithistida) to new orders.

**Figure 5:**
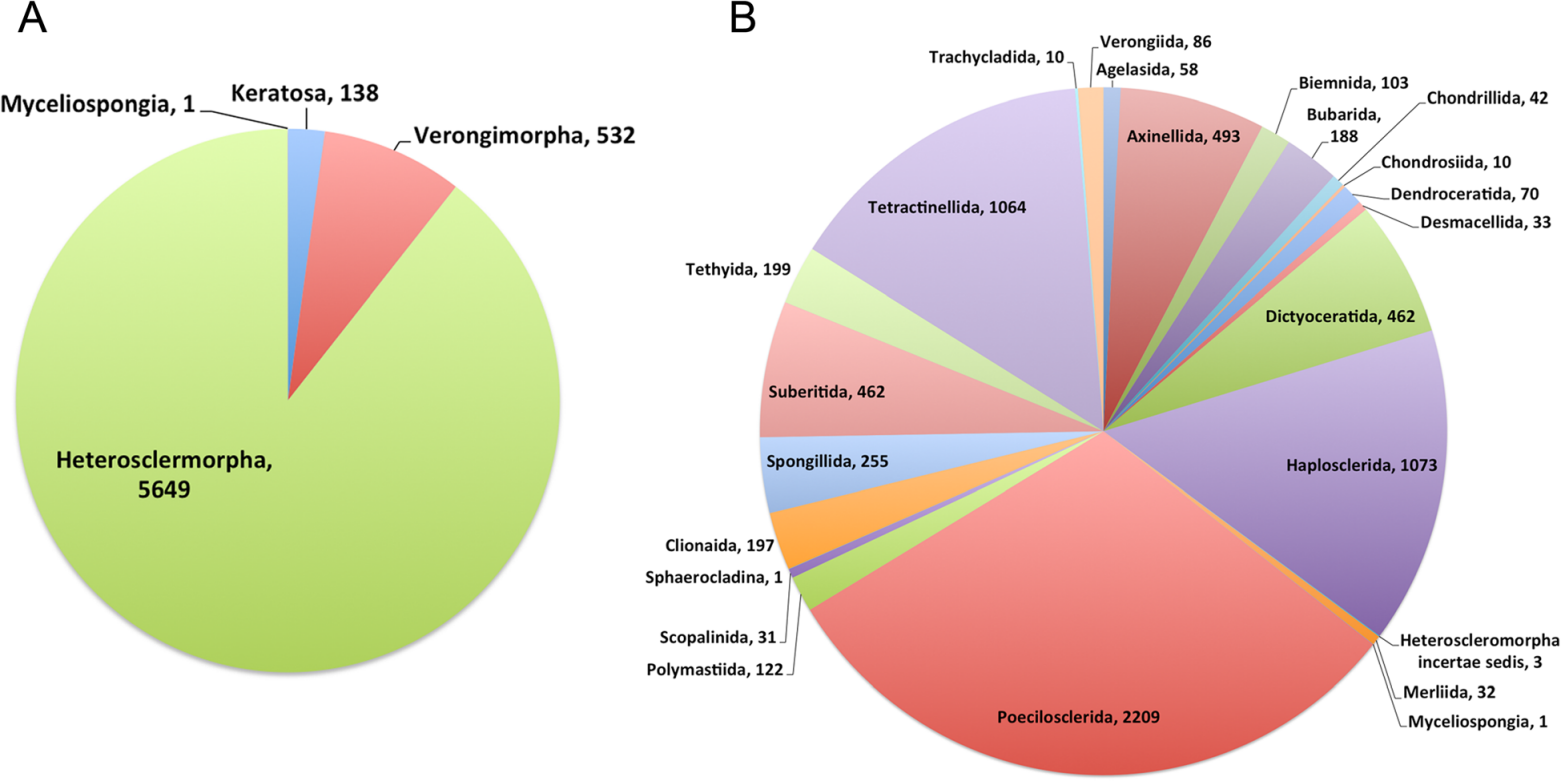
**Pie charts showing the importance of Demospongiae subclasses and orders in terms of number of species. A.** Demospongiae subclasses. **B.** The 22 Demospongiae orders from the revised classification, in alphabetical order. Numbers of species are estimates obtained from the World Porifera Database http://www.marinespecies.org/porifera/ (accessed on the 17th of October 2014).

When the classification changes, so does the importance of the different groups in terms of species numbers; these numbers are reviewed for the various orders in Figure 5. According to SP, Poecilosclerida was the largest order in term of species: over 2,630 species [4, accessed on the 17th of October 2014]. By reassigning five families Poecilosclerida “loses” about 421 species (essentially Raspailiidae). However the revised Poecilosclerida remains the largest order with 2,209 species. The second largest is the revised Haplosclerida with 1,073 species, and the third largest is Tetractinellida (including 11 former lithistid families) with 1,064 species ([4], accessed on the 17th of October 2014).

The overall aim of this paper is to begin to resolve the growing discrepancy between the classification presented in SP and the body of evidence from molecular phylogenetic studies. Doing so, we hope to convince end-users to 1) abandon the use of artificial groups, and to 2) use the new/resurrected names proposed here when referring to the new Demospongiae clades. This updated classification will undoubtedly facilitate communication between end-users, reduce taxonomically biased results, and ultimately provide a better understanding of Demospongiae evolutionary history. We should however keep in mind that the groupings we propose are new phylogenetic hypotheses that will be challenged by future systematic research. Hypotheses regarding sponge phylogenetic relationships will continue to change with the description and sequencing of new species, use of new datasets and improvements in phylogenetic reconstruction methods. New phylogenetic hypotheses will undoubtedly involve further changes to the classification. In other words, absolute nomenclatural stability in a rank-based system is impossible and name changes simply reflect the regular growth of phylogenetic knowledge and understanding [78]. This is certainly frustrating for the many end-users of the sponge classification, but it is a reality that we have to accept and understand if we want our research to rely on updated taxonomic grounds and avoid reaching misleading conclusions. The good news is that end-users and non-sponge specialists can now rely on a large choice of biodiversity web based databases. The WPD is currently the most complete, easily and regularly updated database thanks to a large editorial committee.

Yes, the *Systema Porifera* classification published in 2002 is already partly out of date but we should keep in mind that SP still represents a milestone for sponge researchers, due to its rigorous approach of defining terminal taxa (based on objective evidence from the type species of each genus), and the richness of taxonomic and morphological information it contains. As was certainly the case with SP, we hope the following proposal will stimulate fruitful taxonomic research. In particular, we hope this proposal will help researchers to refocus and revisit clades with a more integrative taxonomic approach [3], combining top-down and bottom-up phylogenetic strategies [3]. Many of these new groupings require clear morphological diagnoses, we hope this classification will help to reveal hidden and overlooked synapomorphies in the various datasets that sponge biologists now have at their disposal. We invite and welcome comments on our proposal, as well as any suggestions for additional changes.

## Appendix

**Proposal for Heteroscleromorpha genera assignments (except Haplosclerida) in their respective orders and families of the revised classification.** This Table 1 lists the Heteroscleromorpha genera (in alphabetical order) with their current assignment in the World Porifera Database http://www.marinespecies.org/porifera/ (accessed on the 17th of October 2014). It does not include the marine haplosclerids as no changes are proposed for this group. For each of the genera, we give the order and family assignment within the framework of the revised classification. Some of these assignments are tentative and may change. We have included the molecular markers that exist for the type species: *CO1* (*cytochrome oxidase subunit 1*, usually the Folmer fragment), *18S* (*ribosomal small subunit*), *28S* (*ribosomal large subunit*), *ITS* (*internal transcribed spacer*), *AGL11* (*aspargine-linked glycosylation 11 protein*), ESTs (Expressed Sequence Tags), mt (mitochondrial genome) and tpm (transcriptome). We have also included absence/presence (yes/no) of molecular markers available for non-type species.

**Table 1:** **Listing of Heteroscleromorpha genera in alphabetical order**

**Genus**	**Order**	**Family**	**New order (Suborder)**	**Family**	**Genes, type species**	**Genes, non-type**
***Aaptos***	Hadromerida	Suberitidae	Suberitida	Suberitidae	no	yes
***Abyssocladia***	Poecilosclerida	Cladorhizidae	Poecilosclerida	Cladorhizidae	no	yes
***Acalle***	Haplosclerida	Metaniidae	Spongillida	Metaniidae	no	no
***Acanthancora***	Poecilosclerida	Hymedesmiidae	Poecilosclerida	Hymedesmiidae	18S	no
***Acanthella***	Halichondrida	Dictyonellidae	Bubarida	Dictyonellidae	18S, 28S, COI	yes
***Acantheurypon***	Poecilosclerida	Raspailiidae	Poecilosclerida	Poecilosclerida *incertae sedis*	18S, 28S, COI	yes
***Acanthochaetetes***	Hadromerida	Acanthochaetetidae	Clionaida	Acanthochaetetidae	no	yes
***Acanthoclada***	Halichondrida	Heteroxyidae	Axinellida	Heteroxyidae	no	no
***Acanthopolymastia***	Hadromerida	Polymastiidae	Polymastiida	Polymastiidae	no	no
***Acanthorhabdus***	Poecilosclerida	Acarnidae	Poecilosclerida	Acarnidae	no	no
***Acanthostylotella***	Poecilosclerida	Raspailiidae	Agelasida	Agelasidae	18S, 28S	no
***Acanthotetilla***	Spirophorida	Tetillidae	Tetractinellida (Spirophorina)	Tetillidae	no	yes
***Acanthotriaena***	Astrophorida	Pachastrellidae	Tetractinellida (Astrophorina)	Pachastrellidae	no	no
***Acanthotylotra***	Haplosclerida	Spongillina *incertae sedis*	Spongillida	Spongillida *incertae sedis*	no	no
***Acarnus***	Poecilosclerida	Acarnidae	Poecilosclerida	AcarnidaeCP	16S, 18S, 28S	no
***Acheliderma***	Poecilosclerida	Acarnidae	Poecilosclerida	Acarnidae	no	no
***Aciculites***	‘Lithistida’	Scleritodermidae	Tetractinellida (Spirophorina)	Scleritodermidae	no	yes
***Adreus***	Hadromerida	Hemiasterellidae	Tethyida	Hemiasterellidae	18S, 28S, COI	yes
***Agelas***	Agelasida	Agelasidae	Agelasida	Agelasidae	28S, COI	yes
***Alectona***	Astrophorida	Thoosidae	Tetractinellida (Astrophorina)	Thoosidae	COI, 28S	no
***Alloscleria***	Halichondrida	Heteroxyidae	Axinellida	Heteroxyidae	no	no
***Amorphinopsis***	Halichondrida	Halichondriidae	Suberitida	Halichondriidae	18S, 28S, COI, EF-1	yes
***Amphiastrella***	Poecilosclerida	Iotrochotidae	Poecilosclerida	Iotrochotidae	no	no
***Amphibleptula***	‘Lithistida’	Scleritodermidae	Tetractinellida (Spirophorina)	Scleritodermidae	no	no
***Amphilectus***	Poecilosclerida	Esperiopsidae	Poecilosclerida	Esperiopsidae	no	yes
***Amphinomia***	Poecilosclerida	Echinodictyinae	Poecilosclerida	Echinodictyinae	18S, 28S	no
***Amphitethya***	Spirophorida	Tetillidae	Tetractinellida (Spirophorina)	Tetillidae	no	yes
***Anaderma***	‘Lithistida’	Pleromidae	Tetractinellida (Astrophorina)	Pleromidae	no	no
***Ancorella***	Astrophorida	Pachastrellidae	Tetractinellida (Astrophorina)	Pachastrellidae	no	no
***Ancorina***	Astrophorida	Ancorinidae	Tetractinellida (Astrophorina)	Ancorinidae	no	yes
***Anheteromeyenia***	Haplosclerida	Spongillidae	Spongillida	Spongillidae	no	no
***Anisocrella***	Poecilosclerida	Crellidae	Poecilosclerida	Crellidae	no	no
***Annulastrella***	Astrophorida	Theneidae	Tetractinellida (Astrophorina)	Theneidae	no	yes
***Antho***	Poecilosclerida	Ophlitaspongiinae	Poecilosclerida	Microcionidae	18S, 28S	yes
***Anthotethya***	Hadromerida	Tethyidae	Tethyida	Tethyidae	no	no
***Arabescula***	‘Lithistida’	Lithistida *incertae sedis*	*incertae sedis*		no	no
***Artemisina***	Poecilosclerida	Ophlitaspongiinae	Poecilosclerida	Microcionidae	no	yes
***Asbestopluma***	Poecilosclerida	Cladorhizidae	Poecilosclerida	Cladorhizidae	no	yes
***Asteropus***	Astrophorida	Ancorinidae	Tetractinellida (Astrophorina)	Ancorinidae	no	yes
***Astrosclera***	Agelasida	Astroscleridae	Agelasida	Astroscleridae	28S,COI,18S, ITS1-5.8S-ITS2-28S	no
***Astrotylus***	Hadromerida	Polymastiidae	Polymastiida	Polymastiidae	no	no
***Atergia***	Hadromerida	Polymastiidae	Polymastiida	Polymastiidae	18S	no
***Auletta***	Halichondrida	Axinellidae	Bubarida	Bubaridae^1^	no	yes
***Aulospongus***	Poecilosclerida	Raspailiidae	Axinellida	Raspailiidae	no	yes
***Awhiowhio***	‘Lithistida’	Corallistidae	Tetractinellida (Astrophorina)	Corallistidae	no	no
***Axechina***	Poecilosclerida	Raspailiidae	Axinellida	Raspailiidae	18S, 28S, COI	no
***Axinella***	Halichondrida	Axinellidae	Axinellida	Axinellidae	18S, 28S	yes
***Axinyssa***	Halichondrida	Halichondriidae	Bubarida	Dictyonellidae	18S, 28S	yes
***Axos***	Hadromerida	Hemiasterellidae	Tethyida	Hemiasterellidae	28S, COI	yes
***Baikalospongia***	Haplosclerida	Lubomirskiidae	Spongillida	Lubomirskiidae	COI, cox2-ATP6, 18S, ITS1-5.8S-ITS2-28S	yes
***Balliviaspongia***	Haplosclerida	Spongillina *incertae sedis*	Spongillida	Spongillida *incertae sedis*	no	no
***Batzella***	Poecilosclerida	Chondropsidae	Poecilosclerida	Chondropsidae	no	no
***Biemna***	Poecilosclerida	Desmacelliae	Biemnida	Biemnidae	18S, 28S, COI	yes
***Brachiaster***	Astrophorida	Pachastrellidae	Tetractinellida (Astrophorina)	Pachastrellidae	no	no
***Bubaris***	Halichondrida	Bubaridae	Bubarida	Bubaridae	no	yes
***Burtonitethya***	Hadromerida	Tethyidae	Tethyida	Tethyidae	no	no
***Callipelta***	‘Lithistida’	Neopeltidae	Tetractinellida (Astrophorina)	Neopeltidae	no	yes
***Calthropella***	Astrophorida	Calthropellidae	Tetractinellida (Astrophorina)	Calthropellidae	no	yes
***Caminella***	Astrophorida	Geodiidae	Tetractinellida (Astrophorina)	Geodiidae	no	yes
***Caminus***	Astrophorida	Geodiidae	Tetractinellida (Astrophorina)	Geodiidae	COI	no
***Cantabrina***	Poecilosclerida	Echinodictyinae	Poecilosclerida	Echinodictyinae	no	no
***Caulospongia***	Hadromerida	Suberitidae	Suberitida	Suberitidae	no	yes
***Celtodoryx***	Poecilosclerida	Coelosphaeridae	Poecilosclerida	Coelosphaeridae	no (contaminant)	no
***Ceratoporella***	Agelasida	Astroscleridae	Agelasida	Astroscleridae	COI, ITS1-5.8S	no
***Ceratopsion***	Poecilosclerida	Raspailiidae	Axinellida	Raspailiidae	no	yes
***Cerbaris***	Halichondrida	Bubaridae	Bubarida	Bubaridae	no	no
***Cercicladia***	Poecilosclerida	Cladorhizidae	Poecilosclerida	Cladorhizidae	no	no
***Cervicornia***	Hadromerida	Clionaidae	Clionaida	Clionaidae	18S, 28S	no
***Chaetodoryx***	Poecilosclerida	Coelosphaeridae	Poecilosclerida	Coelosphaeridae	no	no
***Characella***	Astrophorida	Pachastrellidae	Tetractinellida (Astrophorina)	(Astrophorina) *incertae sedis*	no	yes
***Chelotropella***	Astrophorida	Ancorinidae	Tetractinellida (Astrophorina)	Ancorinidae	no	no
***Chondrocladia***	Poecilosclerida	Cladorhizidae	Poecilosclerida	Cladorhizidae	no	yes
***Chondropsis***	Poecilosclerida	Chondropsidae	Poecilosclerida	Chondropsidae	no	yes
***Cinachyra***	Spirophorida	Tetillidae	Tetractinellida (Spirophorina)	Tetillidae	COI, 28S, 18S	yes
***Cinachyrella***	Spirophorida	Tetillidae	Tetractinellida (Spirophorina)	Tetillidae	no	yes
***Ciocalapata***	Halichondrida	Halichondriidae	Suberitida	Halichondriidae	no	no
***Ciocalypta***	Halichondrida	Halichondriidae	Suberitida	Halichondriidae	18S, 28S, COI	yes
***Cladorhiza***	Poecilosclerida	Cladorhizidae	Poecilosclerida	Cladorhizidae	no	yes
***Cladothenea***	Astrophorida	Theneidae	Tetractinellida (Astrophorina)	Theneidae	no	no
***Clathria***	Poecilosclerida	Microcioninae	Poecilosclerida	Microcionidae	no	yes
***Cliona***	Hadromerida	Clionaidae	Clionaida	Clionaidae	18S, 28S, COI	yes
***Clionaopsis***	Hadromerida	Clionaidae	Clionaida	Clionaidae	no	no
***Cliothosa***	Hadromerida	Clionaidae	Clionaida	Clionaidae	no	no
***Coelocarteria***	Poecilosclerida	Isodictyidae	Poecilosclerida	Isodictyidae	28S	no
***Coelodischela***	Poecilosclerida	Guitarridae	Poecilosclerida	Guitarridae	no	no
***Coelosphaera***	Poecilosclerida	Coelosphaeridae	Poecilosclerida	Coelosphaeridae	no	no
***Collectella***	‘Lithistida’	Lithistida *incertae sedis*	Tetractinellida (Astrophorina)	Theonellidae	no	no
***Columnitis***	Hadromerida	Tethyidae	Tethyida	Tethyidae	no	no
***Corallistes***	‘Lithistida’	Corallistidae	Tetractinellida (Astrophorina)	Corallistidae	no	yes
***Cornulella***	Poecilosclerida	Acarnidae	Poecilosclerida	Acarnidae	no	yes
***Cornulum***	Poecilosclerida	Acarnidae	Poecilosclerida	Acarnidae	no	no
***Cortispongilla***	Haplosclerida	Malawispongiidae	Spongillida	Malawispongiidae	COI, 18S, 5.8S-ITS2-28S	no
***Corvoheteromeyenia***	Haplosclerida	Spongillidae	Spongillida	Spongillidae	no	no
***Corvomeyenia***	Haplosclerida	Metaniidae	Spongillida	Metaniidae	no	yes
***Corvospongilla***	Haplosclerida	Spongillidae	Spongillida	Spongillidae	no	no
***Costifer***	‘Lithistida’	Isoraphiniidae	Tetractinellida (Astrophorina)	Isoraphiniidae	no	no
***Crambe***	Poecilosclerida	Crambeidae	Poecilosclerida	Crambeidae	18S, 28S	yes
***Craniella***	Spirophorida	Tetillidae	Tetractinellida (Spirophorina)	Tetillidae	no	yes
***Crella***	Poecilosclerida	Crellidae	Poecilosclerida	Crellidae	18S, 28S, tpm	yes
***Crellastrina***	Poecilosclerida	Crellidae	Poecilosclerida	Crellidae	no	no
***Crellomima***	Poecilosclerida	Crellidae	Poecilosclerida	Crellidae	no	no
***Cryptax***	Halichondrida	Halichondriidae	Suberitida	Halichondriidae	no	no
***Cryptosyringa***	Astrophorida	Ancorinidae	Tetractinellida (Astrophorina)	Ancorinidae	no	no
***Cyamon***	Poecilosclerida	Raspailiidae	Axinellida	Raspailiidae	no	no
***Cyclacanthia***	Poecilosclerida	Latrunculiidae	Poecilosclerida	Latrunculiidae	no	no
***Cymbastela***	Halichondrida	Axinellidae	Bubarida	Dictyonellidae	28S	yes
***Daedalopelta***	‘Lithistida’	Neopeltidae	Tetractinellida (Astrophorina)	Neopeltidae	no	no
***Damiria***	Poecilosclerida	Acarnidae	Poecilosclerida	Acarnidae	no	no
***Damiriopsis***	Poecilosclerida	Myxillidae	Poecilosclerida	Myxillidae	no	no
***Delectona***	Astrophorida	Thoosidae	Tetractinellida (Astrophorina)	Thoosidae	no	no
***Dendoricella***	Poecilosclerida	Dendoricellidae	Poecilosclerida	Dendoricellidae	no	no
***Dercitus***	Astrophorida	Ancorinidae	Tetractinellida (Astrophorina)	Ancorinidae	COI, 18S	yes
***Desmacella***	Poecilosclerida	Desmacellidae	Desmacellida	Desmacellidae	18S, 28S	yes
***Desmacidon***	Poecilosclerida	Desmacididae	Poecilosclerida	Desmacididae	no	no
***Desmanthus***	‘Lithistida’	Desmanthidae	Bubarida	Bubaridae	18S, 28S, COI	no
***Desmapsamma***	Poecilosclerida	Desmacididae	Poecilosclerida	Desmacididae	16S, 18S, 28S, COI	no
***Desmascula***	‘Lithistida’	Azoricidae	Tetractinellida (Spirophorina)	Azoricidae	no	no
***Desmoxya***	Halichondrida	Heteroxyidae	Axinellida	Heteroxyidae	no	no
***Diacarnus***	Poecilosclerida	Podospongiidae	Poecilosclerida	Podospongiidae^3^	18S, 28S, COI	no
***Dictyonella***	Halichondrida	Dictyonellidae	Bubarida	Dictyonellidae	18S, 28S	yes
***Didiscus***	Halichondrida	Heteroxyidae	Axinellida	Raspailiidae	no	yes
***Diplastrella***	Hadromerida	Spirastrellidae	Clionaida	Spirastrellidae	no	yes
***Diplopodospongia***	Poecilosclerida	Podospongiidae	Poecilosclerida	Podospongiidae	no	no
***Discodermia***	‘Lithistida’	Theonellidae	Tetractinellida (Astrophorina)	Theonellidae	no	yes
***Discorhabdella***	Poecilosclerida	Crambeidae	Poecilosclerida	Crambeidae	no	no
***Disyringa***	Astrophorida	Ancorinidae	Tetractinellida (Astrophorina)	Ancorinidae	18S, 28S	no
***Dolichacantha***	Poecilosclerida	Acarnidae	Poecilosclerida	Acarnidae	no	no
***Dosilia***	Haplosclerida	Spongillidae	Spongillida	Spongillidae	no	no
***Dotona***	Hadromerida	Clionaidae	Clionaida	Clionaidae	no	no
***Dragmacidon***	Halichondrida	Axinellidae	Axinellida	Axinellidae	no	yes
***Dragmatella***	Poecilosclerida	Desmacellidae	Desmacellida	Desmacellidae	no	no
***Dragmaxia***	Halichondrida	Axinellidae	Axinellida	Axinellidae	no	yes
***Drulia***	Haplosclerida	Metaniidae	Spongillida	Metaniidae	5.8S-ITS2-28S	no
***Duosclera***	Haplosclerida	Spongillidae	Spongillida	Spongillidae	no	no
***Dyscliona***	Hadromerida	Clionaidae	Clionaida	Clionaidae	no	no
***Echinochalina***	Poecilosclerida	Microcioninae	Poecilosclerida	Microcionidae	no	yes
***Echinoclathria***	Poecilosclerida	Ophlitaspongiinae	Poecilosclerida	Microcionidae	no	yes
***Echinodictyum***	Poecilosclerida	Echinodictyinae	Poecilosclerida	Echinodictyinae	no	yes
***Echinospongilla***	Haplosclerida	Potamolepidae	Spongillida	Potamolepidae	COI, 18S, ITS1-5.8S-ITS2-28S	no
***Echinostylinos***	Poecilosclerida	Phellodermidae	Poecilosclerida	Phellodermidae	no	no
***Ecionemia***	Astrophorida	Ancorinidae	Tetractinellida (Astrophorina)	Ancorinidae	18S	yes
***Ectyonopsis***	Poecilosclerida	Myxillidae	Poecilosclerida	Myxillidae	no	yes
***Ectyoplasia***	Poecilosclerida	Raspailiidae	Axinellida	Raspailiidae	18S, 28S	yes
***Endectyon***	Poecilosclerida	Raspailiidae	Axinellida	Raspailiidae	no	yes
***Eospongilla***	Haplosclerida	Palaeospongillidae	Spongillida	Palaeospongillidae	no	no
***Ephydatia***	Haplosclerida	Spongillidae	Spongillida	Spongillidae	COI, 18S, 5.8S-ITS2-28S, mt, ESTs	yes
***Epipolasis***	Halichondrida	Axinellidae	Axinellida	Axinellidae	no	yes
***Erylus***	Astrophorida	Geodiidae	Tetractinellida (Astrophorina)	Geodiidae	COI, 28S	yes
***Esperiopsis***	Poecilosclerida	Esperiopsidae	Poecilosclerida	Esperiopsidae	no	no
***Euchelipluma***	Poecilosclerida	Guitarridae	Poecilosclerida	Guitarridae	no	no
***Eunapius***	Haplosclerida	Spongillidae	Spongillida	Spongillidae	COI, ITS2, 18S	yes
***Eurypon***	Poecilosclerida	Raspailiidae	Axinellida	Raspailiidae	no	yes
***Exsuperantia***	‘Lithistida’	Phymaraphiniidae	Tetractinellida (Astrophorina)	Phymaraphiniidae	no	yes
***Fangophilina***	Spirophorida	Tetillidae	Tetractinellida (Spirophorina)	Tetillidae	no	yes
***Fibulia***	Poecilosclerida	Dendoricellidae	Poecilosclerida	Dendoricellidae	no	no
***Forcepia***	Poecilosclerida	Coelosphaeridae	Poecilosclerida	Coelosphaeridae	no	yes
***Gastrophanella***	‘Lithistida’	Siphonidiidae	Tetractinellida (Spirophorina)	Siphonidiidae	no	no
***Geodia***	Astrophorida	Geodiidae	Tetractinellida (Astrophorina)	Geodiidae	no	yes
***Goreauiella***	Agelasida	Astroscleridae	Agelasida	Astroscleridae	no	no
***Guitarra***	Poecilosclerida	Guitarridae	Poecilosclerida	Guitarridae	28S	no
***Halichondria***	Halichondrida	Halichondriidae	Suberitida	Halichondriidae	18S, 28S, 5.8S, COI	yes
***Halicnemia***	Halichondrida	Heteroxyidae	Axinellida	Stelligeridae	no	yes
***Halicometes***	Hadromerida	Tethyidae	Tethyida	Tethyidae	no	no
***Hamacantha***	Poecilosclerida	Hamacanthidae	Merliida	Hamacanthidae	no	no
***Hamigera***	Poecilosclerida	Hymedesmiidae	Poecilosclerida	Hymedesmiidae	18S, 28S	yes
***Hemiasterella***	Hadromerida	Hemiasterellidae	Tethyida	Hemiasterellidae	no	yes
***Hemimycale***	Poecilosclerida	Hymedesmiidae	Poecilosclerida	Hymedesmiidae	18S, 28S	no
***Hemitedania***	Poecilosclerida	Tedaniidae	Poecilosclerida	Tedaniidae	no	no
***Herengeria***	‘Lithistida’	Corallistidae	Tetractinellida (Astrophorina)	Corallistidae	no	no
***Heteromeyenia***	Haplosclerida	Spongillidae	Spongillida	Spongillidae	no	no
***Heterorotula***	Haplosclerida	Spongillidae	Spongillida	Spongillidae	no	yes
***Heteroxya***	Halichondrida	Heteroxyidae	Axinellida	Heteroxyidae	no	no
***Higginsia***	Halichondrida	Heteroxyidae	Axinellida	Stelligeridae	no	yes
***Hispidopetra***	Agelasida	Astroscleridae	Agelasida	Astroscleridae	no	no
***Histodermella***	Poecilosclerida	Coelosphaeridae	Poecilosclerida	Coelosphaeridae	no	no
***Holopsamma***	Poecilosclerida	Microcioninae	Poecilosclerida	Microcionidae	no	yes
***Holoxea***	Astrophorida	Ancorinidae	Tetractinellida (Astrophorina)	Ancorinidae	no	yes
***Homaxinella***	Hadromerida	Suberitidae	Suberitida	Suberitida *incertae sedis*	no	yes
***Homophymia***	‘Lithistida’	Neopeltidae	Tetractinellida (Astrophorina)	Neopeltidae	no	yes
***Houssayella***	Haplosclerida	Metaniidae	Spongillida	Metaniidae	no	no
***Hymedesmia***	Poecilosclerida	Hymedesmiidae	Poecilosclerida	Hymedesmiidae	no	yes
***Hymenancora***	Poecilosclerida	Myxillidae	Poecilosclerida	Myxillidae	no	no
***Hymeniacidon***	Halichondrida	Halichondriidae	Suberitida	Halichondriidae	18S, 28S, COI	yes
***Hymeraphia***	Poecilosclerida	Raspailiidae	Axinellida	Raspailiidae	18S, 28S	yes
***Hymerhabdia***	Agelasida	Hymerhabdiidae	Agelasida	Hymerhabdiidae	18S, 28S, COI	no
***Hymetrochota***	Poecilosclerida	Iotrochotidae	Poecilosclerida	Iotrochotidae	no	no
***Inflatella***	Poecilosclerida	Coelosphaeridae	Poecilosclerida	Coelosphaeridae	18S	no
***Iophon***	Poecilosclerida	Acarnidae	Poecilosclerida	Acarnidae	no	yes
***Iotroata***	Poecilosclerida	Iotrochotidae	Poecilosclerida	Iotrochotidae	no	no
***Iotrochopsamma***	Poecilosclerida	Iotrochotidae	Poecilosclerida	Iotrochotidae	no	no
***Iotrochota***	Poecilosclerida	Iotrochotidae	Poecilosclerida	Iotrochotidae	18S, 28S, mt	no
***Isabella***	‘Lithistida’	Corallistidae	Tetractinellida (Astrophorina)	Corallistidae	no	no
***Isodictya***	Poecilosclerida	Isodictyidae	Poecilosclerida	Isodictyidae	no	yes
***Janulum***	Poecilosclerida	Raspailiidae	Haplosclerida	Phloeodictyidae	18S	no
***Jaspis***	Astrophorida	Ancorinidae	Tetractinellida (Astrophorina)	Ancorinidae	no	yes
***Jereicopsis***	‘Lithistida’	Azoricidae	Tetractinellida (Spirophorina)	Azoricidae	no	no
***Johannesia***	Halichondrida	Halichondriidae	Suberitida	Halichondriidae	no	no
***Julavis***	Halichondrida	Heteroxyidae	Axinellida	Heteroxyidae	no	no
***Kaliapsis***	‘Lithistida’	Phymaraphiniidae	Tetractinellida (Astrophorina)	Phymaraphiniidae	no	no
***Kirkpatrickia***	Poecilosclerida	Hymedesmiidae	Poecilosclerida	Hymedesmiidae	no	no
***Lamellomorpha***	Astrophorida	Pachastrellidae	Tetractinellida (Astrophorina)	(Astrophorina) *incertae sedis*	18S	no
***Laminospongia***	Halichondrida	Halichondriidae	Suberitida	Halichondriidae	no	no
***Latrunculia***	Poecilosclerida	Latrunculiidae	Poecilosclerida	Latrunculiidae	no	yes
***Laxotethya***	Hadromerida	Tethyidae	Tethyida	Tethyidae	28S	no
***Leiodermatium***	‘Lithistida’	Azoricidae	Tetractinellida (Spirophorina)	Azoricidae	no	no
***Lepidosphaera***	Poecilosclerida	Coelosphaeridae	Poecilosclerida	Coelosphaeridae	no	no
***Lepidothenea***	‘Lithistida’	Phymaraphiniidae	Tetractinellida (Astrophorina)	Phymaraphiniidae	no	no
***Leptosastra***	Hadromerida	Hemiasterellidae	Tethyida	Hemiasterellidae	no	no
***Liosina***	Halichondrida	Dictyonellidae	Tethyida	Hemiasterellidae	28S, COI	no
***Lipastrotethya***	Halichondrida	Dictyonellidae	Bubarida	Dictyonellidae	no	no
***Lissodendoryx***	Poecilosclerida	Coelosphaeridae	Poecilosclerida	Coelosphaeridae	no	yes
***Lithobactrum***	‘Lithistida’	Siphonidiidae	Tetractinellida (Spirophorina)	Siphonidiidae	no	no
***Lithochela***	Poecilosclerida	Crambeidae	Poecilosclerida	Crambeidae	no	no
***Lithoplocamia***	Poecilosclerida	Raspailiidae	Poecilosclerida	Raspailiidae	no	no
***Lollipocladia***	Poecilosclerida	Cladorhizidae	Poecilosclerida	Cladorhizidae	no	no
***Lubomirskia***	Haplosclerida	Lubomirskiidae	Spongillida	Lubomirskiidae	65 sequences, mt, ESTs	yes
***Lutetiospongilla***	Haplosclerida	Palaeospongillidae	Spongillida	Palaeospongillidae	no	no
***Macandrewia***	‘Lithistida’	Macandrewiidae	Tetractinellida (Astrophorina)	Macandrewiidae	no	no
***Makedia***	Haplosclerida	Spongillina *incertae sedis*	Spongillida	Spongillida *incertae sedis*	no	no
***Malawispongia***	Haplosclerida	Malawispongiidae	Spongillida	Malawispongiidae	no	no
***Manihinea***	‘Lithistida’	Theonellidae	Tetractinellida (Astrophorina)	Theonellidae	18S	no
***Megaciella***	Poecilosclerida	Acarnidae	Poecilosclerida	Acarnidae	no	no
***Melonanchora***	Poecilosclerida	Myxillidae	Poecilosclerida	Myxillidae	no	no
***Melophlus***	Astrophorida	Geodiidae	Tetractinellida (Astrophorina)	Geodiidae	no	yes
***Merlia***	Poecilosclerida	Merliidae	Merliida	Merliidae	16S, 18S, 28S	no
***Metania***	Haplosclerida	Metaniidae	Spongillida	Metaniidae	no	no
***Metschnikowia***	Haplosclerida	Metschnikowiidae	Spongillida	Metschnikowiidae	no	no
***Microscleroderma***	‘Lithistida’	Scleritodermidae	Tetractinellida (Spirophorina)	Scleritodermidae	no	yes
***Microtylostylifer***	Poecilosclerida	Desmacellidae	Desmacellida	Desmacellidae	no	no
***Microxistyla***	Halichondrida	Heteroxyidae	Axinellida	Heteroxyidae	no	no
***Monanchora***	Poecilosclerida	Crambeidae	Poecilosclerida	Crambeidae	28S, COI	yes
***Monocrepidium***	Halichondrida	Bubaridae	Bubarida	Bubaridae	no	no
***Mycale***	Poecilosclerida	Mycalidae	Poecilosclerida	Mycalidae	no	yes
***Myrmekioderma***	Halichondrida	Heteroxyidae	Axinellida	Heteroxyidae	18S, 28S, COI	yes
***Myxilla***	Poecilosclerida	Myxillidae	Poecilosclerida	Myxillidae	28S	yes
***Myxodoryx***	Poecilosclerida	Hymedesmiidae	Poecilosclerida	Hymedesmiidae	no	no
***Neamphius***	Astrophorida	Thoosidae	Tetractinellida (Astrophorina)	(Astrophorina) *incertae sedis*	COI, 28S	yes
***Negombata***	Poecilosclerida	Podospongiidae	Poecilosclerida	Podospongiidae	18S, COI	yes
***Negombo***	Halichondrida	Heteroxyidae	Axinellida	Heteroxyidae	no	no
***Neoaulaxinia***	‘Lithistida’	Phymatellidae	Tetractinellida (Astrophorina)	Phymatellidae	no	no
***Neocladia***	Poecilosclerida	Cladorhizidae	Poecilosclerida	Cladorhizidae	no	yes
***Neofibularia***	Poecilosclerida	Desmacellidae	Biemnida	Biemnidae	18S, 28S, COI	yes
***Neopelta***	‘Lithistida’	Neopeltidae	Tetractinellida (Astrophorina)	Neopeltidae	no	no
***Neophrissospongia***	‘Lithistida’	Corallistidae	Tetractinellida (Astrophorina)	Corallistidae	no	yes
***Neopodospongia***	Poecilosclerida	Podospongiidae	Poecilosclerida	Podospongiidae	no	yes
***Neoschrammeniella***	‘Lithistida’	Corallistidae	Tetractinellida (Astrophorina)	Corallistidae	no	no
***Neosiphonia***	‘Lithistida’	Phymatellidae	Tetractinellida (Astrophorina)	Phymatellidae	no	no
***Nethea***	Astrophorida	Pachastrellidae	Tetractinellida (Astrophorina)	Pachastrellidae	no	yes
***Nucleotethya***	Hadromerida	Tethyidae	Tethyida	Tethyidae	no	no
***Nudospongilla***	Haplosclerida	Spongillidae	Spongillida	Spongillidae	no	yes
***Ochridaspongia***	Haplosclerida	Malawispongiidae	Spongillida	Malawispongiidae	no	no
***Ohridospongilla***	Haplosclerida	Spongillina *incertae sedis*	Spongillida	Spongillida *incertae sedis*	no	yes
***Oncosclera***	Haplosclerida	Potamolepidae	Spongillida	Potamolepidae	no	yes
***Onotoa***	Hadromerida	Placospongiidae	Clionaida	Placospongiidae	no	no
***Ophiraphidites***	Halichondrida	Axinellidae	Bubarida	Bubaridae	no	no
***Ophlitaspongia***	Poecilosclerida	Ophlitaspongiinae	Poecilosclerida	Microcionidae	16S, 18S	no
***Oxytethya***	Hadromerida	Tethyidae	Tethyida	Tethyidae	no	no
***Pachastrella***	Astrophorida	Pachastrellidae	Tetractinellida (Astrophorina)	Pachastrellidae	no	yes
***Pachydictyum***	Haplosclerida	Malawispongiidae	Spongillida	Malawispongiidae	COI, 18S, ESTs	yes
***Pachymatisma***	Astrophorida	Geodiidae	Tetractinellida (Astrophorina)	Geodiidae	COI, 28S, 18S, ITS1-5.8S-ITS2	yes
***Pachyrotula***	Haplosclerida	Spongillidae	Spongillida	Spongillidae	no	no
***Palaeospongilla***	Haplosclerida	Palaeospongillidae	Spongillida	Palaeospongillidae	no	no
***Pandaros***	Poecilosclerida	Raspailiidae	Axinellida	Raspailiidae	COI	no
***Paracornulum***	Poecilosclerida	Acarnidae	Poecilosclerida	Acarnidae	COI	no
***Paradesmanthus***	‘Lithistida’	Desmanthidae	Bubarida	Bubaridae	no	no
***Parahigginsia***	Halichondrida	Heteroxyidae	Axinellida	Heteroxyidae	no	no
***Pararhaphoxya***	Halichondrida	Axinellidae	Bubarida	Bubaridae	no	yes
***Paratetilla***	Spirophorida	Tetillidae	Tetractinellida (Spirophorina)	Tetillidae	no	yes
***Paratimea***	Hadromerida	Stelligeridae	Axinellida	Stelligeridae	no	yes
***Pectispongilla***	Haplosclerida	Spongillidae	Spongillida	Spongillidae	no	no
***Penares***	Astrophorida	Geodiidae	Tetractinellida (Astrophorina)	Geodiidae	5.8S-ITS2-28S	yes
***Penicillus***	Hadromerida	Polymastiidae	Polymastiida	Polymastiidae	no	no
***Petromica***	‘Lithistida’	Desmanthidae	*incertae sedis*		no	yes
***Phakellia***	Halichondrida	Axinellidae	Bubarida	Bubaridae	18S, 28S, COI	yes
***Phakettia***	Halichondrida	Dictyonellidae	Bubarida	Dictyonellidae	no	yes
***Phelloderma***	Poecilosclerida	Phellodermidae	Poecilosclerida	Phellodermidae	no	yes
***Phlyctaenopora***	Poecilosclerida	Mycalidae	Poecilosclerida	Mycalidae	no	no
***Phorbas***	Poecilosclerida	Hymedesmiidae	Poecilosclerida	Hymedesmiidae	18S	yes
***Phoriospongia***	Poecilosclerida	Chondropsidae	Poecilosclerida	Chondropsidae	no	no
***Phycopsis***	Halichondrida	Axinellidae	Bubarida	Bubaridae	no	yes
***Pione***	Hadromerida	Clionaidae	Clionaida	Clionaidae	no	yes
***Pipestela***	Halichondrida	Axinellidae	Bubarida	Bubaridae	no	no
***Placospherastra***	Hadromerida	Placospongiidae	Clionaida	Placospongiidae	no	no
***Placospongia***	Hadromerida	Placospongiidae	Clionaida	Placospongiidae	18S, COI	yes
***Plakidium***	‘Lithistida’	Lithistida *incertae sedis*	*incertae sedis*		no	no
***Pleroma***	‘Lithistida’	Pleromidae	Tetractinellida (Astrophorina)	Pleromidae	no	no
***Plicatellopsis***	Hadromerida	Suberitidae	Suberitida	Suberitidae	no	no
***Plocamiancora***	Poecilosclerida	Myxillidae	Poecilosclerida	Myxillidae	no	yes
***Plocamione***	Poecilosclerida	Raspailiidae	Poecilosclerida	Raspailiidae	no	no
***Plocamionida***	Poecilosclerida	Hymedesmiidae	Poecilosclerida	Hymedesmiidae	18S, 28S, COI	yes
***Podospongia***	Poecilosclerida	Podospongiidae	Poecilosclerida	Podospongiidae	no	no
***Poecillastra***	Astrophorida	Vulcanellidae	Tetractinellida (Astrophorina)	Vulcanellidae	COI, 28S	yes
***Polymastia***	Hadromerida	Polymastiidae	Polymastiida	Polymastiidae	no	yes
***Pomelia***	‘Lithistida’	Scleritodermidae	Tetractinellida (Spirophorina)	Scleritodermidae	no	no
***Poritella***	‘Lithistida’	Lithistida *incertae sedis*	*incertae sedis*		no	no
***Potamolepis***	Haplosclerida	Potamolepidae	Spongillida	Potamolepidae	no	yes
***Potamophloios***	Haplosclerida	Potamolepidae	Spongillida	Potamolepidae	no	no
***Pottsiela***	Haplosclerida	Spongillidae	Spongillida	Spongillidae	no	no
***Pozziella***	Poecilosclerida	Hamacanthidae	Merliida	Hamacanthidae	no	no
***Prosuberites***	Hadromerida	Suberitidae	Agelasida	Hymerhabdiidae	28S	yes
***Proteleia***	Hadromerida	Polymastiidae	Polymastiida	Polymastiidae	18S	no
***Protosuberites***	Hadromerida	Suberitidae	Suberitida	Suberitidae	no	yes
***Psammastra***	Astrophorida	Ancorinidae	Tetractinellida (Astrophorina)	Ancorinidae	no	no
***Psammochela***	Poecilosclerida	Myxillidae	Poecilosclerida	Myxillidae	no	no
***Psammoclema***	Poecilosclerida	Chondropsidae	Poecilosclerida	Chondropsidae	no	yes
***Pseudohalichondria***	Poecilosclerida	Hymedesmiidae	Poecilosclerida	Hymedesmiidae	no	no
***Pseudospongosorites***	Hadromerida	Suberitidae	Suberitida	Suberitidae	tpm	no
***Pseudosuberites***	Hadromerida	Suberitidae	Suberitida	Suberitidae	no	yes
***Pseudotrachya***	Hadromerida	Polymastiidae	Polymastiida	Polymastiidae	no	no
***Ptilocaulis***	Halichondrida	Axinellidae	Axinellida	Raspailiidae	18S, 28S, mt	yes
***Pyloderma***	Poecilosclerida	Dendoricellidae	Poecilosclerida	Dendoricellidae	no	no
***Quasillina***	Hadromerida	Polymastiidae	Polymastiida	Polymastiidae	18S	no
***Racekiela***	Haplosclerida	Spongillidae	Spongillida	Spongillidae	no	no
***Racodiscula***	‘Lithistida’	Theonellidae	Tetractinellida (Astrophorina)	Theonellidae	no	no
***Radiella***	Hadromerida	Polymastiidae	Polymastiida	Polymastiidae	no	no
***Radiospongilla***	Haplosclerida	Spongillidae	Spongillida	Spongillidae	no	yes
***Raspaciona***	Poecilosclerida	Raspailiidae	Axinellida	Raspailiidae	18S, 28S, COI	no
***Raspailia***	Poecilosclerida	Raspailiidae	Axinellida	Raspailiidae	no	yes
***Reidispongia***	‘Lithistida’	Phymatellidae	Tetractinellida (Astrophorina)	Phymatellidae	no	no
***Reniochalina***	Halichondrida	Axinellidae	Axinellida	Raspailiidae	18S, 28S	yes
***Rezinkovia***	Haplosclerida	Lubomirskiidae	Spongillida	Lubomirskiidae	18S-5.8S-ITS2-28S, mt	yes
***Rhabdastrella***	Astrophorida	Ancorinidae	Tetractinellida (Astrophorina)	Geodiidae	no	yes
***Rhabderemia***	Poecilosclerida	Rhabderemiidae	Biemnida	Rhabderemiidae	no	yes
***Rhabdeurypon***	Poecilosclerida	Raspailiidae	Axinellida	Raspailiidae	no	no
***Rhabdobaris***	Halichondrida	Bubaridae	Bubarida	Bubaridae	no	no
***Rhaphidhistia***	Hadromerida	Trachycladidae	Trachycladida	Trachycladidae	no	no
***Rhaphoxya***	Halichondrida	Dictyonellidae	Bubarida	Dictyonellidae	no	no
***Rhizaxinella***	Hadromerida	Suberitidae	Suberitida	Suberitidae	no	yes
***Ridleia***	Hadromerida	Polymastiidae	Polymastiida	Polymastiidae	no	no
***Rotuloplocamia***	Poecilosclerida	Iotrochotidae	Poecilosclerida	Iotrochotidae	no	no
***Samus***	Spirophorida	Samidae	Tetractinellida (Spirophorina)	Samidae	no	no
***Sanidastra***	Haplosclerida	Spongillidae	Spongillida	Spongillidae	no	no
***Sarcomella***	Halichondrida	Halichondriidae	Suberitida	Halichondriidae	no	no
***Saturnospongilla***	Haplosclerida	Spongillidae	Spongillida	Spongillidae	no	no
***Sceptrella***	Poecilosclerida	Latrunculiidae	Poecilosclerida	Latrunculiidae	no	yes
***Sceptrintus***	Poecilosclerida	Podospongiidae	Poecilosclerida	Podospongiidae	no	no
***Scleritoderma***	‘Lithistida’	Scleritodermidae	Tetractinellida (Spirophorina)	Scleritodermidae	no	no
***Scolopes***	Hadromerida	Clionaidae	Clionaida	Clionaidae	no	no
***Scopalina***	Halichondrida	Scopalinidae	Scopalinida	Scopalinidae	18S, 28S, COI	yes
***Semisuberites***	Poecilosclerida	Esperiopsidae	Poecilosclerida	Esperiopsidae	no	no
***Setidium***	‘Lithistida’	Scleritodermidae	Tetractinellida (Spirophorina)	Scleritodermidae	no	no
***Sigmaxinella***	Poecilosclerida	Desmacellidae	Biemnida	Biemnidae	no	yes
***Sigmeurypon***	Poecilosclerida	Ophlitaspongiinae	Poecilosclerida	Microcionidae	no	no
***Sigmosceptrella***	Poecilosclerida	Podospongiidae	Poecilosclerida	Podospongiidae	no	no
***Siliquariaspongia***	‘Lithistida’	Theonellidae	Tetractinellida (Astrophorina)	Theonellidae	no	no
***Siphonidium***	‘Lithistida’	Siphonidiidae	Tetractinellida (Spirophorina)	Siphonidiidae	no	no
***Sollasella***	Poecilosclerida	Raspailiidae	Axinellida	Raspailiidae	no	yes
***Spanioplon***	Poecilosclerida	Hymedesmiidae	Poecilosclerida	Hymedesmiidae	18S, 28S	no
***Sphaerotylus***	Hadromerida	Polymastiidae	Polymastiida	Polymastiidae	no	yes
***Spheciospongia***	Hadromerida	Clionaidae	Clionaida	Clionaidae	18S	yes
***Spinospongilla***	Haplosclerida	Malawispongiidae	Spongillida	Malawispongiidae	no	no
***Spinularia***	Hadromerida	Polymastiidae	Polymastiida	Polymastiidae	no	no
***Spirasigma***	Spirophorida	Spirasigmidae	Tetractinellida (Spirophorina)	Spirasigmidae	no	no
***Spirastrella***	Hadromerida	Spirastrellidae	Clionaida	Spirastrellidae	no	yes
***Spirorhabdia***	Poecilosclerida	Crellidae	Poecilosclerida	Crellidae	no	no
***Spiroxya***	Hadromerida	Clionaidae	Clionaida	Clionaidae	no	no
***Spongilla***	Haplosclerida	Spongillidae	Spongillida	Spongillidae	COI,18S, 28S, ITS1-5.8S-ITS2-28S, tpm	yes
***Spongosorites***	Halichondrida	Halichondriidae	Suberitida	Halichondriidae	no	yes
***Stelletta***	Astrophorida	Ancorinidae	Tetractinellida (Astrophorina)	Ancorinidae	COI, 28S, 18S	yes
***Stellettinopsis***	Astrophorida	Ancorinidae	Tetractinellida (Astrophorina)	Ancorinidae	no	yes
***Stelligera***	Hadromerida	Stelligeridae	Axinellida	Stelligeridae	18S, 28S, COI	yes
***Stellitethya***	Hadromerida	Tethyidae	Tethyida	Tethyidae	no	yes
***Stelodoryx***	Poecilosclerida	Myxillidae	Poecilosclerida	Myxillidae	no	no
***Sterrastrolepis***	Haplosclerida	Potamolepidae	Spongillida	Potamolepidae	no	no
***Stratospongilla***	Haplosclerida	Spongillidae	Spongillida	Spongillidae	no	no
***Stromatospongia***	Agelasida	Astroscleridae	Agelasida	Astroscleridae	no	no
***Strongylacidon***	Poecilosclerida	Chondropsidae	Poecilosclerida	Chondropsidae	no	yes
***Strongylamma***	Poecilosclerida	Tedaniidae	Poecilosclerida	Tedaniidae	no	no
***Strongylodesma***	Poecilosclerida	Latrunculiidae	Poecilosclerida	Latrunculiidae	no	no
***Stryphnus***	Astrophorida	Ancorinidae	Tetractinellida (Astrophorina)	Ancorinidae	no	yes
***Stylissa***	Halichondrida	Scopalinidae	Scopalinida	Scopalinidae	18S, 28S, COI	yes
***Stylocordyla***	Hadromerida	Stylocordylidae	Suberitida	Stylocordylidae	COI (submitted)	no
***Suberites***	Hadromerida	Suberitidae	Suberitida	Suberitidae	18S, 28S, mt, ESTs	yes
***Sulcastrella***	‘Lithistida’	Desmanthidae	*incertae sedis*		no	no
***Svenzea***	Halichondrida	Scopalinidae	Scopalinida	Scopalinidae	18S, 28S, COI	yes
***Swartschewskia***	Haplosclerida	Lubomirskiidae	Spongillida	Lubomirskiidae	COI, 18S, 5.8S-ITS2-28S, mt	no
***Tectitethya***	Hadromerida	Tethyidae	Tethyida	Tethyidae	no	yes
***Tedania***	Poecilosclerida	Tedaniidae	Poecilosclerida	Tedaniidae	no	yes
***Tedaniphorbas***	Poecilosclerida	Acarnidae	Poecilosclerida	Acarnidae	no	no
***Tentorina***	Spirophorida	Spirasigmidae	Tetractinellida (Spirophorina)	Spirasigmidae	no	no
***Tentorium***	Hadromerida	Polymastiidae	Polymastiida	Polymastiidae	18S	no
***Terpios***	Hadromerida	Suberitidae	Suberitida	Suberitida *incertae sedis*	no	yes
***Tethya***	Hadromerida	Tethyidae	Tethyida	Tethyidae	18S, 28S, COI	yes
***Tethyastra***	Hadromerida	Tethyidae	Tethyida	Tethyidae	no	no
***Tethycometes***	Hadromerida	Tethyidae	Tethyida	Tethyidae	no	no
***Tethyopsis***	Astrophorida	Ancorinidae	Tetractinellida (Astrophorina)	Ancorinidae	no	yes
***Tethyspira***	Halichondrida	Dictyonellidae	Axinellida	Raspailiidae	18S, 28S, COI	no
***Tethytimea***	Hadromerida	Tethyidae	Tethyida	Tethyidae	no	yes
***Tetilla***	Spirophorida	Tetillidae	Tetractinellida (Spirophorina)	Tetillidae	no	yes
***Tetrapocillon***	Poecilosclerida	Guitarridae	Poecilosclerida	Guitarridae	no	yes
***Thenea***	Astrophorida	Theneidae	Tetractinellida (Astrophorina)	Theneidae	COI, 28S	yes
***Theonella***	‘Lithistida’	Theonellidae	Tetractinellida (Astrophorina)	Theonellidae	COI, 28S, 18S	yes
***Thoosa***	Astrophorida	Thoosidae	Tetractinellida (Astrophorina)	Thoosidae	no	no
***Thrinacophora***	Poecilosclerida	Raspailiidae	Axinellida	Raspailiidae	no	yes
***Thrombus***	Astrophorida	Thrombidae	Tetractinellida (Astrophorina)	Thrombidae	28S	no
***Timea***	Hadromerida	Timeidae	Tethyida	Tethyidae	no	yes
***Topsentia***	Halichondrida	Halichondriidae	Suberitida	Suberitida *incertae sedis*	no	yes
***Trachostylea***	Poecilosclerida	Raspailiidae	Axinellida	Raspailiidae	no	no
***Trachycladus***	Hadromerida	Trachycladidae	Trachycladida	Trachycladidae	18S, 28S	yes
***Trachyteleia***	Hadromerida	Polymastiidae	Polymastiida	Polymastiidae	no	no
***Tribrachium***	Astrophorida	Ancorinidae	Tetractinellida (Astrophorina)	Ancorinidae	no	no
***Trikentrion***	Poecilosclerida	Raspailiidae	Axinellida	Raspailiidae	no	yes
***Triptolemma***	Astrophorida	Pachastrellidae	Tetractinellida (Astrophorina)	Pachastrellidae	no	yes
***Trochospongilla***	Haplosclerida	Spongillidae	Spongillida	Spongillidae	no	yes
***Tsitsikamma***	Poecilosclerida	Latrunculiidae	Poecilosclerida	Latrunculiidae	18S, COI	no
***Tylexocladus***	Hadromerida	Polymastiidae	Polymastiida	Polymastiidae	no	no
***Ulosa***	Poecilosclerida	Esperiopsidae	Poecilosclerida^2^	Esperiopsidae^2^	no	yes
***Umborotula***	Haplosclerida	Spongillidae	Spongillida	Spongillidae	no	no
***Uritaia***	Halichondrida	Halichondriidae	Suberitida	Halichondriidae	no	no
***Uruguaya***	Haplosclerida	Potamolepidae	Spongillida	Potamolepidae	no	no
***Uruguayella***	Haplosclerida	Spongillidae	Spongillida	Spongillidae	no	no
***Vetulina***	‘Lithistida’	Vetulinidae	Sphaerocladina	Vetulinidae	18S	yes
***Volzia***	Hadromerida	Clionaidae	Clionaida	Clionaidae	no	no
***Vosmaeria***	Halichondrida	Halichondriidae	Suberitida	Halichondriidae	28S	no
***Vulcanella***	Astrophorida	Vulcanellidae	Tetractinellida (Astrophorina)	Vulcanellidae	no	yes
***Waltherarndtia***	Poecilosclerida	Raspailiidae	Axinellida	Raspailiidae	no	no
***Weberella***	Hadromerida	Polymastiidae	Polymastiida	Polymastiidae	no	no
***Wigginsia***	Poecilosclerida	Acarnidae	Poecilosclerida	Acarnidae	no	no
***Willardia***	Hadromerida	Acanthochaetetidae	Clionaida	Acanthochaetetidae	no	no
***Xenospongia***	Hadromerida	Tethyidae	Tethyida	Tethyidae	18S, 28S	no
***Yucatania***	Astrophorida	Thrombidae	Tetractinellida (Astrophorina)	Thrombidae	no	no
***Zyzzya***	Poecilosclerida	Acarnidae	Poecilosclerida	Acarnidae	no	yes

^1^ One sequence of *Auletta* in Erpenbeck et al. [39] groups with *Acanthella* and *Phakellia ventilabrum.*

^2^ *28S* sequences of *Ulosa digitata* suggest that this genus could belong to the Suberitida, as it groups with Halichondriidae [5].

^3^ Redmond et al. [10] show *D. spinipoculum* (type species) clustering with Raspailiidae whilst *D. bismarckensis* groups with Podospongiidae. It is possible that the *18S* sequence of *D. spinipoculum* in Redmond et al. [10] is a result of contamination and therefore we retain *Diacarnus* in Podospongiidae.
